# Data-driven modelling of a gene regulatory network for cell fate decisions in the growing limb bud

**DOI:** 10.15252/msb.20145882

**Published:** 2015-07-14

**Authors:** Manu Uzkudun, Luciano Marcon, James Sharpe

**Affiliations:** 1 EMBL-CRG Systems Biology Program, Centre for Genomic Regulation (CRG), Universitat Pompeu Fabra (UPF)Barcelona, Spain; 2 Institucio Catalana de Recerca i Estudis Avancats (ICREA)Barcelona, Spain

**Keywords:** gene regulatory networks, limb development, morphogen, patterning, reverse-engineering

## Abstract

Parameter optimization coupled with model selection is a convenient approach to infer gene regulatory networks from experimental gene expression data, but so far it has been limited to single cells or static tissues where growth is not significant. Here, we present a computational study in which we determine an optimal gene regulatory network from the spatiotemporal dynamics of gene expression patterns in a complex 2D growing tissue (non-isotropic and heterogeneous growth rates). We use this method to predict the regulatory mechanisms that underlie proximodistal (PD) patterning of the developing limb bud. First, we map the expression patterns of the PD markers Meis1, Hoxa11 and Hoxa13 into a dynamic description of the tissue movements that drive limb morphogenesis. Secondly, we use reverse-engineering to test how different gene regulatory networks can interpret the opposing gradients of fibroblast growth factors (FGF) and retinoic acid (RA) to pattern the PD markers. Finally, we validate and extend the best model against various previously published manipulative experiments, including exogenous application of RA, surgical removal of the FGF source and genetic ectopic expression of Meis1. Our approach identifies the most parsimonious gene regulatory network that can correctly pattern the PD markers downstream of FGF and RA. This network reveals a new model of PD regulation which we call the “crossover model”, because the proximal morphogen (RA) controls the distal boundary of Hoxa11, while conversely the distal morphogens (FGFs) control the proximal boundary.

## Introduction

**Figure 1:**
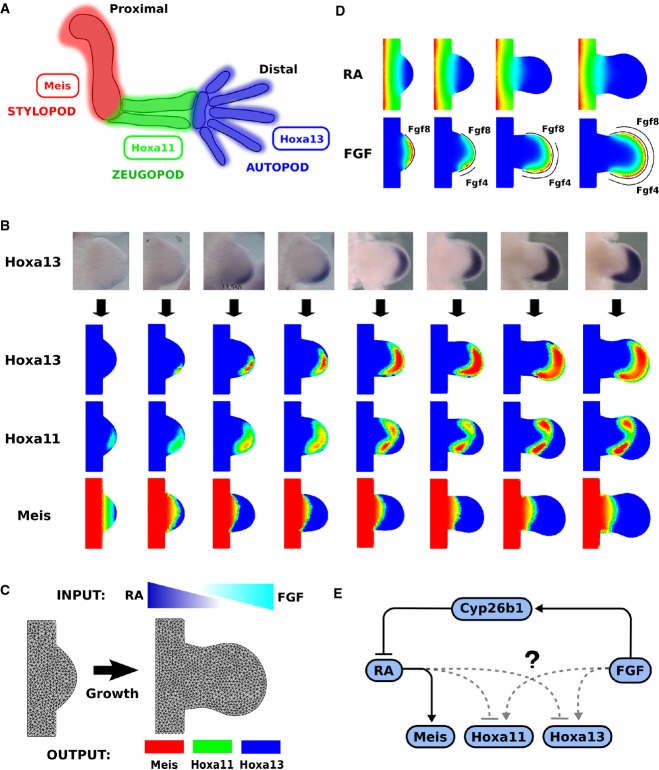
A realistic data-driven model for PD patterning in the limb bud Each PD skeletal element expresses specific genes. Meis1 and Meis 2 are stylopod markers, Hoxa11 is the zeugopod marker, and Hoxa13 is the autopod marker.

The vertebrate limb is a classical model system to study the specification of a growing organ during development. In the mouse, limb development starts with the outgrowth of the limb bud from the lateral plate mesoderm, which in just two days is able to form the main skeletal elements present in the adult limb. The limb skeleton is divided into three main proximodistal (PD) segments: the stylopod (upper arm/leg), the zeugopod (lower arm/leg) and the autopod (hand/foot) (Fig1A). The three segments are specified in a proximal-to-distal sequence, first the stylopod, then the zeugopod and finally the autopod (Tabin and Wolpert, 2007; Towers *et al*, 2012). The regulatory mechanism that specifies the three PD segments sequentially is not yet fully understood (Tabin and Wolpert, 2007; Roselló-Díez *et al*, 2014).

The process of mapping experimental gene expression data of Meis, Hoxa11 and Hoxa13 to the 2D limb bud model.

The general regulatory model uses the RA and FGF morphogen gradients as inputs and should explain the expression patterns of the PD markers as outputs: Meis, Hoxa11 and Hoxa13 over time and space.

An example of the simulated morphogen gradients of RA and FGF. The source of FGF signal (curved black lines) is combined from the FGF8 expression pattern, which is uniform along the AER, and the FG4 expression pattern, which is initially expressed in a small posterior region which is later expanded anteriorly.

Solid interactions describe the “upstream” part of the circuit, which we take as given. The main hypothetical interactions to be explored in this study are shown as the dashed lines—the regulation of the Hox genes.

During the development of the limb bud, a set of specific transcription factors marks the developing PD segments. Currently, the best known PD markers are homeobox genes: Meis1 and Meis2 (stylopod), Hoxa11 (zeugopod) and Hoxa13 (autopod) (Nelson *et al*, 1996; Tabin and Wolpert, 2007; Mercader *et al*, 2009) (Fig1A). In the initial stages of limb development (around E9.25 stage), Meis1 and Meis2 are expressed across the entire limb bud. Successively, around E10.0, they are downregulated in the distal region where Hoxa11 is activated (Fig1B). Finally, around E10.5, Hoxa13 starts to be expressed in a small distal posterior region and expands anteriorly and proximally with a simultaneous shrinking of Hoxa11, which then maintains a mutually exclusive pattern with Hoxa13 (Nelson *et al*, 1996; Tabin and Wolpert, 2007; Mercader *et al*, 2009). The question of how these three molecular zones are controlled by upstream signals has traditionally been split into two categories of model: (i) those in which cells make decisions by measuring the duration of exposure to a signal, or alternatively, (ii) those in which the decision is based on the strengths or levels of a signal.

A classical model to explain the specification of the limb PD axis is the progress zone model (PZM). This model proposes that the progressive specification of the PD segments is based on a timing mechanism which is active in a distal region, called the progress zone (PZ), that is under the influence of the apical ectodermal ridge (AER) (Summerbell 1974). As the limb grows, cells exit the progress zone and assume different fates depending on the amount of time they received the “distalizing” fibroblast growth factor (FGF) signals coming from the AER (Niswander *et al* 1993; Fallon *et al* 1994). Under this hypothesis, it is the duration of exposure to FGF which determines how distal the cell fate will be—a longer duration specifies a more distal fate. It is important to note that this hypothesis does not specify the mechanism by which time is measured. It could be counting temporal oscillations (e.g. the cell cycle), or it could simply be the slow accumulation of a factor in the cells. Four different FGFs are expressed in the mouse AER, and although their precise role in PD patterning is still debated (Mariani *et al*, 2008; Yu and Ornitz, 2008), it is clear that they also play a major role in the physical outgrowth of the limb. FGF8 is expressed along all the AER and is the most essential for correct growth of the limb bud (Mariani *et al*, 2008). Fgf4,9,17 are functionally redundant and are expressed initially in a posterior region and then expand anteriorly (Mariani *et al*, 2008).

A more recent model of PD patterning is the two-signal model (2SM) (Mercader *et al*, 2000). This model explains the specification of the stylopod by a mechanism based on two opposing signals: a distal FGF signal coming from the AER and a proximal signal coming from the body flank (Mercader *et al*, 2000; Cooper *et al*, 2011; Roselló-Díez *et al*, 2011). Experimental evidence suggests that retinoic acid (RA) plays the role of the proximal signal (Mercader *et al*, 2000; Cooper *et al*, 2011; Roselló-Díez *et al*, 2011) although a consensus on the importance of RA in PD patterning has not yet been reached (Cunningham and Duester, 2015). RA is synthesized in the lateral plate mesoderm (LPM) by the enzyme RALDH2 and diffuses into the mesenchyme (Yashiro *et al*, 2004). FGF signalling in the distal tissue promotes the expression of Cyp26b1, an enzyme that degrades RA to inactive forms (Probst *et al*, 2011)—thereby creating a gradient of RA along the PD axis (Yashiro *et al*, 2004). In the proximal tissue, RA upregulates the expression of the stylopod markers Meis1 and Meis2, while in the distal tissue, FGF signalling appears to downregulate them, probably through the promotion of Cyp26b1 (Mercader *et al*, 2000; Cooper *et al*, 2011; Roselló-Díez *et al*, 2011). In the 2SM, the stylopod–zeugopod boundary is thus explained as the tipping point between the proximal influence of RA and the distal influence of FGF, rer than as a timing mechanism.

Recently, the idea of timing has again been revived (Roselló-Díez *et al*, 2014) in a study which showed that prematurely exposing distal tissue to the signalling environment typical for late distal tissue (i.e. low RA and high FGF) is unable to induce precocious expression of the distal marker Hoxa13. Moreover, it showed that reducing RA could only induce extra Hoxa13 expression after the developmental stage when Hoxa13 has been naturally activated. The fact that this could be overcome by promoting chromatin opening suggested that an epigenetic timing mechanism might also be involved (Roselló-Díez *et al*, 2014). It should be noted, however, that this proposal was not exactly the same as the PZM, as the authors were able to rule out a simple temporal integration of FGF signalling.

In summary, the main alternative models for this process still focus on the distinction between measuring signal duration versus measuring signalling levels. Here, we use an approach combining parameter optimization and model selection to investigate these different hypotheses in an unbiased way. First, we map the gene expression of the PD markers over space and time into an accurate model of limb morphogenesis (Marcon *et al*, 2011). Then, we reverse-engineer the optimal gene network that controls the patterning of the PD markers by testing different networks that act downstream of the proposed FGF and RA gradients. Our method makes no assumption on which mechanistic concept underlies PD patterning (i.e. whether the system measures signalling levels, or measures time). We instead employ a step-by-step approach, starting from a basic network that describes the known regulation between the stylopod marker Meis and the RA/FGF signals (Mercader *et al*, 2000; Yashiro *et al*, 2004; Cooper *et al*, 2011; Roselló-Díez *et al*, 2011). We then test different ways in which Hoxa11 and Hoxa13 could be regulated by a combination of RA and FGF. We fit the different regulatory networks by inferring the optimal parameter values that can better reproduce the experimental wild-type expression patterns of Meis, Hoxa11 and Hoxa13.

This systems biology approach allows us to identify which is the simplest network that can explain the known experimental data on the three PD markers. Finally, we use the model to investigate how the network relates to the different conceptual models that have been proposed to explain the specification of the PD segments. This allows to us determine to which extent a model based on measuring levels, versus measuring time, underlies PD patterning. Achieving this in a system with dynamically moving tissue does not alter the basic objective function (i.e. the method by which we calculate the difference between simulated and experimental data), but it allows us to find the model which explains the whole sequence of gene regulatory events despite the constantly changing shape of the growing tissue during this period.

## Results

### Optimizing the null hypotheses: RA- or FGF-dominant models

To study different hypothetical PD patterning mechanisms, we simulated gene regulatory networks on a realistic growing model of limb development. This was done by using an accurate 2D numerical description of limb morphogenesis (Marcon *et al*, 2011) that has been previously generated by combining a morphometric analysis of limb shapes (Boehm *et al*, 2011) with clonal fate mapping data (Arques *et al*, 2007). The growth of the limb is represented by a 2D triangular mesh with anisotropic growth distortions, which is fully remeshed at 1-hour intervals over a 36-hour period to maintain high quality of the mesh throughout the simulation (Marcon *et al*, 2011). We used this framework to investigate how well different gene regulatory networks can pattern the PD markers from stage mE10:09 to stage mE11:12. To simulate the dynamics of the relative concentrations of the PD markers and morphogen gradients (FGF and RA), we employed reaction–diffusion partial differential equations (PDEs). These equations are solved on the growing triangular mesh using a finite-volume method. Our software uses precalculated remeshing and handles the redistribution of element contents into the new mesh at 1-h intervals, which is described in detail in Marcon *et al* (2011). The rate of change in the concentration of a given gene product *G* is given by three terms in equation 1: a production term *P*_*G*_, a diffusion term *D*_*G*_ and a linear decay term *λ*_*G*_. The production terms for all molecules vary in time and space. In the case of FGF and RA, these non-uniform production terms are mapped from experimental data, while for the other molecules, production rates are calculated from the regulatory dynamics of the network.

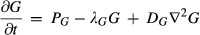
1

To obtain the experimental data needed for the reverse-engineering, we performed whole-mount *in situ* hybridizations (WMISH) of the main PD markers: Meis1, Hoxa11 and Hoxa13, from stage mE10:9 to stage mE11:12. These data are used to constrain the models and to implement the objective function for the optimization process. The Meis1 and Meis2 genes show similar expression patterns and patterning roles, so we chose Meis1 as representative of both. We mapped these data into the computational growth map by converting colour intensity values to relative and approximate molecular concentrations (see Materials and Methods, and Fig1B). *In situ* hybridization is known to be a non-quantitative technique; however, previous studies in *Drosophila* (in a static 1D domain) have shown that successful reverse-engineering of gene regulatory networks does not require knowledge of the absolute concentrations of gene products (Jaeger *et al*, 2004; Crombach *et al*, 2012). Instead, it is the shapes of the gene expression domains which matter. To ensure that a non-quantitative read-out of the expression levels is not a problem for our study, we chose to perform an explicit non-linear sigmoid rescaling of the mapped values—thereby ensuring that the primary information in the data is about the shape of the expression domains rer than levels (see Materials and Methods for more details, and further discussion towards the end of the next results section).

Our next goal was to optimize the parameter values of different gene regulatory networks to reproduce the wild-type gene expression patterns of Meis, Hoxa11 and Hoxa13 by interpreting the RA and FGF gradients (Fig1C). As our model is a high-level one, we use a single variable to represent FGF signalling, even though it is composed of multiple FGF ligands. To take into account the different FGF sources, we calculated FGF production as a combination of two primary sources: (i) the FGF8 pattern is expressed earlier and more uniformly along all the AER and (ii) the patterns of FGF4, 9 and 17 are initiated slightly later with a clear posterior bias and then gradually expand anteriorly to produce more uniform distributions (Fig1D) (Mariani *et al*, 2008). RA in the model is synthesized at a constant rate at the main body of the embryo and diffuses into the limb mesenchyme (Yashiro *et al*, 2004). The simulated FGF and RA gradients at different stages are shown in Fig1D.

As a starting point for the models, we used a simple regulatory network that includes a set of key molecular components and interactions that are well documented in literature. In particular, we considered the basic hypothesis that the distal FGF morphogen counteracts the action of a proximal RA gradient by induction of Cyp26b1, a RA-degrading enzyme (Mercader *et al*, 2000; Yashiro *et al*, 2004; Cooper *et al*, 2011, Roselló-Díez *et al*, 2011; Roselló-Díez *et al*, 2014). Moreover, we considered that the stylopod marker Meis1 (Mercader *et al*, 1999; Capdevila *et al*, 1999) is upregulated directly by RA in the proximal limb bud (Mercader *et al*, 2000). This basic regulatory network describing these interactions is shown as the solid interactions in Fig1E.

Using the simulated gradients, we explored the different ways in which FGF and RA could regulate the expression of the PD markers Meis, Hoxa11 and Hoxa13. How the FGF and RA signals control the distal markers Hoxa11 and Hoxa13 is still a matter of debate (Vargesson *et al*, 2001; Tabin and Wolpert, 2007; Roselló-Díez *et al*, 2014). It is theoretically possible that both of these opposing gradients directly feed into both genes—thus allowing different thresholds of signalling ratio to be encoded independently for each gene. However, since FGF is known to regulate RA (Mercader *et al*, 2000; Yashiro *et al*, 2004; Probst *et al*, 2011), it is also possible that each Hox gene is primarily regulated by one pway or the other. We used our modelling approach to explore the hypothetical scenarios. In each model, the interactions were implemented with Hill functions that capture that main qualitative feature of gene regulation: a cooperative non-linear responses coupled with saturation (Goutelle *et al*, 2008). We define a model to describe the dynamics of FGF(F), RA(R), Cyp26b1(C) and Meis(M), equations (2–5). This is the “upstream” part of the network (mentioned above, Fig1E), whose topology (regulatory circuit) is common to all models tested in this study. *P*_*F*_, *P*_*R*_, *P*_*C*_ and *P*_*M*_ are the corresponding production rates and *λ*_*F*_, *λ*_*R*_, *λ*_*C*_ and *λ*_*M*_ are the corresponding decay rates. *D*_*F*_ and *D*_*R*_ are the diffusion constants for the FGF and RA diffusible molecules, respectively. *k*’s are specific constants of the Hill function relating to a threshold for activation or inhibition, *μ*’s are Hill coefficients describing the steepness of the regulatory response, and *c*_1_ describes the linear strength by which Cyp26b1 degrades RA.

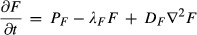
2

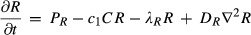
3

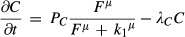
4

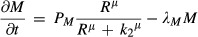
5

The parameters of this system do not represent measurable biochemical constants but rer effective regulatory influences. One exception is the diffusion constants RA and FGF that are obtained from experimental estimates found in literature. For RA, we used a diffusion constant of *D* = 600 μm^2^/min, estimated from experiments in chick wing buds where a bead releasing RA was applied in the anterior wing bud region (Tickle *et al*, 1985). For FGF, we used a diffusion constant of *D* = 100 μm^2^/min, estimated using FRAP measurements in zebra fish (Müller *et al*, 2013).

**Figure 2:**
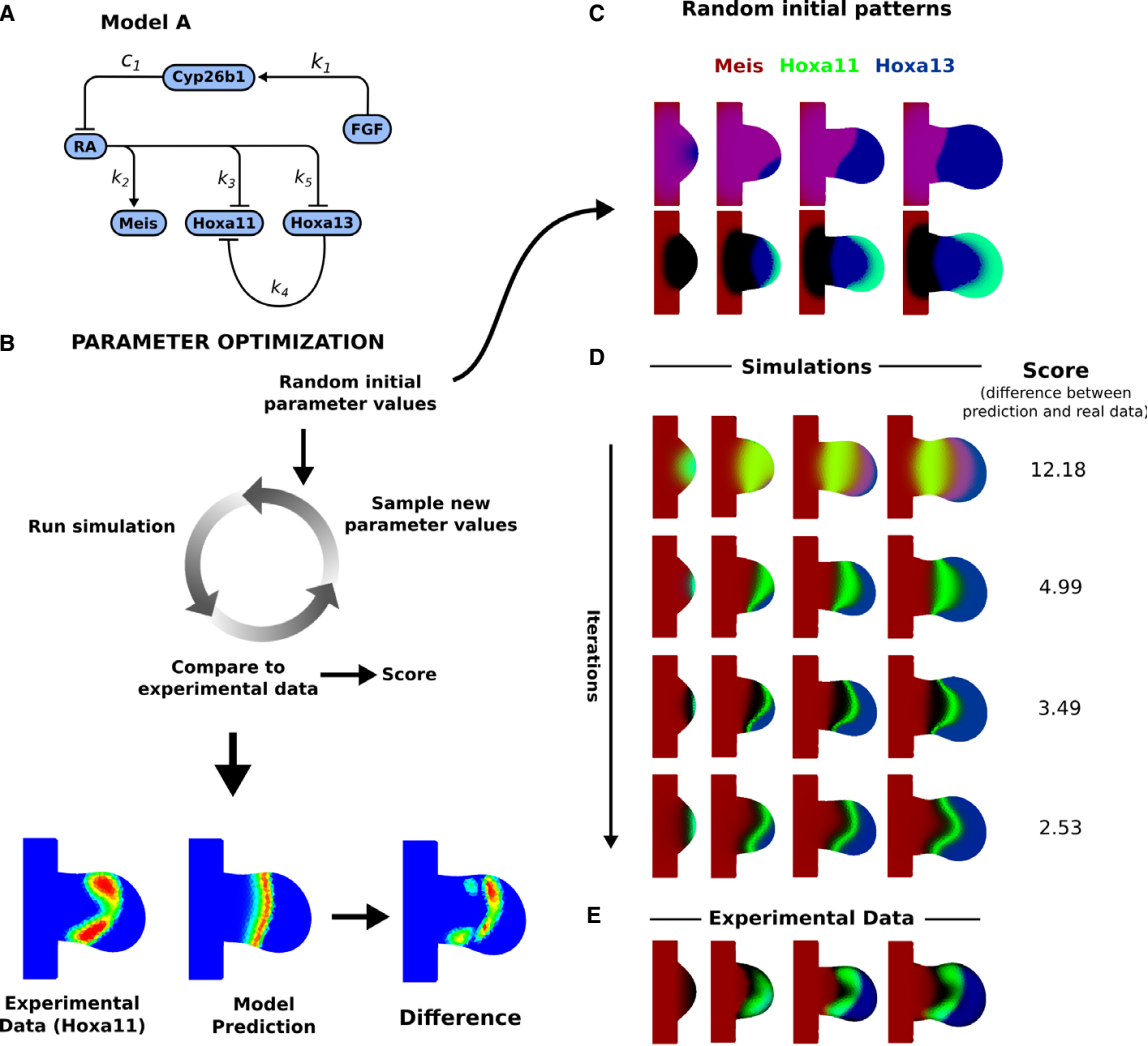
Reverse-engineering (parameter optimization) of a regulatory network in a 2D growing domain An example of a regulatory network with a set of unknown parameters.

Starting from this upstream network, we explored how different models that interpret FGF and RA could regulate the expression of the PD markers Hoxa11 and Hoxa13. These different models correspond to different combinations of the dashed regulatory interactions in Fig1E. Since there are two downstream genes, the minimal models all have two links, and there are consequently four possible minimal models, which we label A–D (and will be described in the subsequent sections one by one). To determine how well each model could theoretically explain the experimental data, we employed a parameter-fitting approach in which the parameters *λ*_*F*_, *λ*_*R*_, *c*_1_, *μ* and the various regulatory *k*’s were optimized to give the best match between the simulated dynamic gene expression patterns and the experimental data. As far as we know, this is the first case of automated reverse-engineering (parameter optimization plus model selection) on a 2D growing/moving tissue. As experimental data, we used wild-type gene expression patterns of Meis, Hoxa11 and Hoxa13 (Fig1B). For each model (e.g. Model A in Fig2A), we employed a gradient descent optimization method (see Materials and Methods) in which the objective function consisted of an automatic scoring method which compared the predicted 2D patterns of each of the three genes with the digitized versions of the wild-type patterns (Fig2B). The goal was not to search only for a good final result, but rer to find networks which recapitulate the entire developmental trajectory of dynamic expression patterns, so pattern scoring was performed for a series of time-points during development (not just the end-point of the simulations). The total score for a given simulation was defined as the sum of the scores for each gene product (Meis, Hoxa11, Hoxa13) and each experimental time-point. The score for a given gene product at a specific experimental time-point was defined as the sum of squared differences for each triangular element 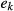 of the experimental and simulated concentrations (equation 6). Rer than the absolute values of concentrations at each position, it is the shapes of the expression domains that matter for the objective function, so we normalized both the experimental and predicted values by a non-linear rescaling process (see Materials and Methods)

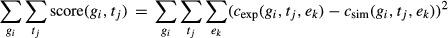
6

The optimization algorithm iteratively finds a parameter set that minimizes the difference between the simulated and experimental gene expression patterns of Meis, Hoxa11 and Hoxa13 at different stages.

Two examples of the simulation using random initial parameter values before optimization.

The gradually improving predicted expression patterns over time and space, for different iterations of the algorithm. Meis is shown in red, Hoxa11 in green, and Hoxa13 in red.

The experimentally mapped gene expression patterns are shown with the same colour code.

For each model, the optimization process was run 27 times, starting from different initial parameter values which were partially random, but selected to be far away from each other in parameter space (see Materials and Methods). This was to ensure that a wide region of parameter space was being explored and to test whether good solutions were converging to the same global optimum, or whether conversely multiple local optima were being found. Two examples run with very different initial parameter values are shown in Fig2C, indicating how far the optimizations start from a successful result. A summary of all initial parameter combinations is given in Supplementary Fig S1 and Supplementary Table S1.

We first tested two simple models based on the idea that both Hoxa11 and Hoxa13 are regulated only by the RA gradient (Roselló-Díez *et al*, 2014) or only by the FGF gradient. In both models, we also include the previously documented inhibition between Hoxa11 by Hoxa13 (Nelson *et al*, 1996; Mercader *et al*, 2009). Since our molecular variables represent relative concentrations (rer than absolute concentrations), both the maximum production rates and the decay rates for Cyp26b1, Meis, Hoxa11 and Hoxa13 were fixed to the arbitrary value of 0.05/min (so that the maximum steady-state concentrations would be 1.0). The strength and speed of each gene regulatory interaction are represented by the steepness of the Hill functions. We used one Hill coefficient *μ* to govern the steepness of all regulatory interactions except for one exception. For the repression of Hoxa11 by Hoxa13, we allowed a distinct Hill coefficient 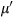, since this repression is experimentally seen to be extremely rapid [the expression patterns of the two genes become quickly mutually exclusive (Nelson *et al*, 1996; Mercader *et al*, 2009)]. Both *μ* and 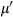 were free parameters to be optimized during the fitting process.

**Figure 3:**
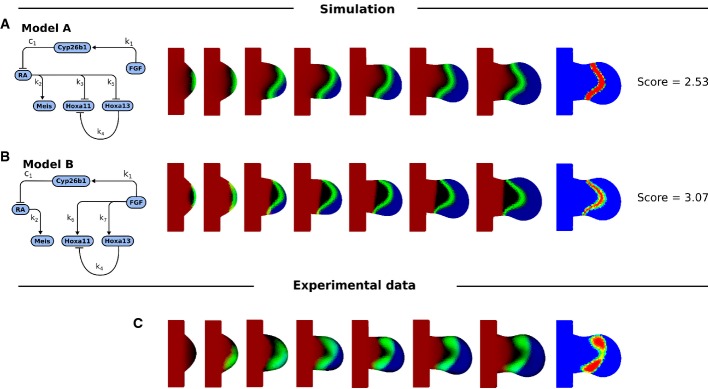
Simulation results of Model A and Model B The regulatory network for Model A, and the simulated expression patterns of Meis shown in red, Hoxa11 in green, and Hoxa13 in red at successive time-points.

In the first model (Model A, see Fig2A), Hoxa11 and Hoxa13 are inhibited by the RA acid gradient and not directly regulated by FGF, as suggested in the most recent study (Roselló-Díez *et al*, 2014). Therefore, the expression of Hoxa11 should start when the RA concentration falls below a certain threshold value, and Hoxa13 should be initiated when the levels further decrease below a second threshold value. This model contains no direct activator of Hoxa11 or Hoxa13, only two different permissive conditions specified by the RA gradient. The regulatory inputs into the Hox genes are given by equations (7, 8) for Hoxa11(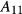) and Hoxa13(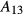), where 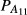 and 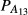 are the corresponding production rates and 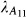 and 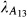 are the corresponding decay rates. *k*_3_, *k*_4_, *k*_5_, *k*_6_ and *k*_7_ are again specific constants for the corresponding Hill functions (see Fig3A).

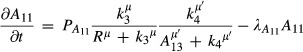
7

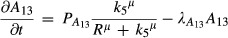
8

The regulatory network Model B, and the simulated expression patterns with the same colour code.

The same time-points as (A) and (B) showing the mapped experimental data. The last column for all rows shows the Hoxa11 pattern at the last time-point, using an intensity colour map to highlight the shape of the expression domain.

In contrast, in the second model (Model B), both Hoxa11 and Hoxa13 are activated by Fgf but not regulated by RA. In equations (9, 10), we describe it is dynamics.

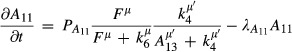
9

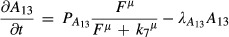
10

We optimized the parameters of both models to fit the wild-type pattern of Meis, Hoxa11 and Hoxa13. An example of the iterative improvements in the gene expression patterns is shown for Model A in Fig2D, which gradually converges to the most similar dynamic sequence of marker patterns. (The optimized values of all parameters for all models discussed in this paper are provided in Supplementary Table S2). After optimization, both Models A and B were able to recapitulate a number of features of the dynamic gene regulation of Hoxa11 and Hoxa13: the general positioning of the domains and the timing of their appearance (Fig3). However, a more detailed examination of the patterns suggested that neither model was able to reproduce certain important qualitative features of the Hoxa11 pattern. In particular, the simulated Hoxa11 domains appeared as a curved stripe which is thicker in the middle than at the top or bottom (i.e. thicker at the centre than at the anterior or posterior ends of the domain). By contrast, the real expression pattern is thinner and weaker in the middle, giving a curved “dumb-bell” type of shape. We thus explored an alternative model which could rectify this problem.

### The value of 2D shape data: the crossover model

**Figure 4:**
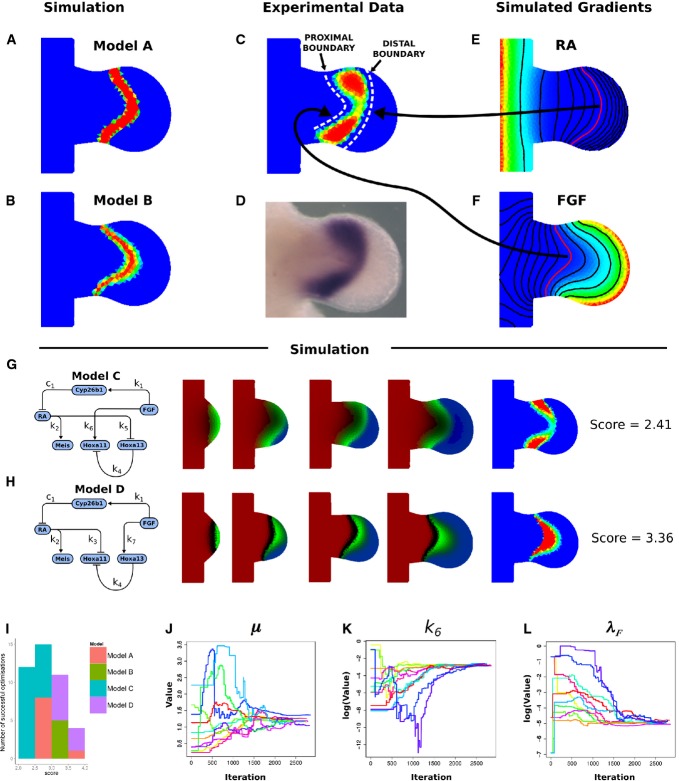
Proximal and distal expression boundaries of Hoxa11 and the crossover model A, B The predicted late expression patterns for Hoxa11 obtained using Model A or Model B.

A detailed analysis of the experimental Hoxa11 pattern revealed that its proximal boundary is more curved than the distal boundary (white dashed lines in Fig4C). Moreover, the central part of the expression pattern is narrower and weaker than the anterior and posterior ends. A possible explanation for the failure of Models A and B to reproduce this expression pattern is that the Hox expression domains in these models are controlled only by one gradient in each case (the RA gradient for Model A and the FGF gradient for Model B). Examination of the shapes of these two gradients showed that they have different spatial profiles. The isoclines for RA (lines which connect all points with the same concentration) were less curved than those for FGF (Fig4E and F). This is because the source of RA is essentially a straight line (the main body of the embryo), while FGFs are produced along a distal curved line that corresponds to the AER.

C–F The real experimental pattern shows quite a different shape—a “dumb-bell” type of shape which is narrower in the middle. The proximal expression boundary is more curved than the distal boundary. Related to this, the shape of the RA gradient (E) is less curved than the shape of the FGF gradient (F).

G, H When Model C is optimized, it produces a dynamical model with a good fit to the experimental data on Hoxa11. By contrast, Model D (H) provides a very poor fit.

I–L When all four models are compared, it is clear that Model C produces the lowest scores, but also achieves successful optimization more frequently than the others. The *x*-axis shows the score, with better results to the left (lower difference values), while the *y*-axis shows the frequency of achieving this score. (J, K, L) show the convergence of free parameters towards optimal regions in parameter space for Model C.

These observations suggested that the FGF gradient may define the more curved proximal boundary of Hoxa11, while the RA gradient may instead define the straighter distal boundary. We explored this hypothesis using another simple model in which the proximal boundary of Hoxa11 was controlled by a positive input from the FGF gradient. This would upregulate Hoxa11 expression on the distal side of a given FGF concentration threshold. However, Hoxa11 is also known to be downregulated distally in an indirect manner by Hoxa13 (Nelson *et al*, 1996; Mercader *et al*, 2009). We therefore considered that if RA had a repressive effect on Hoxa13, it could downregulate Hoxa11 indirectly on the distal part where RA concentrations are lower. We constructed Model C as a hybrid of Models A and B: Hoxa11 activated by FGF and Hoxa13 repressed by RA (Fig4G). We named this the “crossover model” as it has the interesting feature that the *proximal* boundary of Hoxa11 is controlled by the *distal* gradient, and vice versa. The new equations for the regulation of the Hox genes are (11, 12).

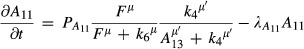
11

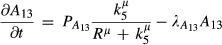
12

To test this hypothesis, we performed the same optimization procedure as before and obtained the simulated expression patterns as shown in Fig4G. The crossover model was indeed better able to recapitulate the shape of the Hoxa11 pattern than the previous models where the regulation was implemented only by one of the two gradients (a difference score of 2.41 compared to 2.53 for Model A, and compare Fig4H with Fig3).

Our hypothesis suggested that the final of the possible minimal models (Model D) would be the worst of all—it should force the distal boundary of Hoxa11 to be more curved than the proximal boundary (the opposite of the observed pattern). When we performed parameter optimization on Model D using equations 13 and 14, it indeed produced the worst fit of all minimal models (a difference score of 3.36).

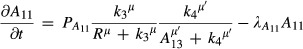
13

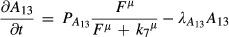
14

The conclusion from optimizing all four basic models was that Model C was the best. However, we wanted to confirm this conclusion as strongly as possible, with a series of extra optimizations and simulations. Firstly, as mentioned above, *in situ* hybridization is known to be a non-quantitative technique. To boost our confidence that the results did not depend on the non-linear rescaling of expression levels, we altered this sigmoid rescaling of values (both experimental and simulated) so that the spatial patterns were slightly changed (see Supplementary Fig S2). This was designed to be equivalent to “underdeveloping” the colour reaction of the *in situ* (or overexposing the image) which could have non-uniform impacts on the final distribution of values. We then reperformed the 27 parameter optimizations for each of the four models, and although the absolute score values changed, Model C still produced the best fit. This confirmed that the important constraint from the data is not the absolute values, but rer the shape of the expression domains. Secondly, to explore whether we have enough data to constrain the model, we removed half of the time-points of the experimental data for Hoxa11 and Hoxa13, and reran the optimization for Model C. If overfitting was a problem, then halving the number of data points would reveal an instability in the fitted parameters, but instead the resulting simulation was indistinguishable from the original result (see Supplementary Fig S3). Thirdly, we analysed the convergence of parameter values during the multiple optimization runs. As can be seen in Fig4J–L, successful results always resulted in parameters converging to the same position of parameter space, providing confidence that a global optimum has been found. (Further analysis of parameter values was performed at the end of the study on Model F.)

Lastly, we chose to optimize a new model (Model *X*_0_), which rer than being one of the simplest topologies, represents the “most complex” topology; that is, it contains all the possible four regulatory links between the upstream nodes RA/FGF and the downstream nodes Hox genes (the grey dashed links in Fig1E). We optimized this “super-model” (Supplementary Fig S4) and then tested it by removing each regulatory link one by one (thereby testing a series of intermediate models, which we called *X*_1_−*X*_4_). In agreement with all results described above, the links which are most important to maintaining a good score are indeed the two links of Model C. Indeed, when we remove both of the unimportant links, we have recreated Model C and the resulting pattern is almost as good as when Model C was optimized directly.

The ranking of the four minimal models (A–D) could be seen not only by their difference scores, but also by how frequently they managed to converge at all, with the best model (C) managing to converge far more often than the next best model (19 out of 27 optimization runs, as compared to 9/27 for Model A). These results are summarized in Fig4I. It should also be emphasized that all four minimal models have the same number of free parameters (they all have two regulatory links between RA/FGF and the Hox genes), so Model C’s success is not just a question of having more degrees of freedom. Overall, these results highlight the power of analysing 2D gene expression patterns as a means to distinguish between different alternative models. All four models are equally capable of recapitulating the correct order of genes along the PD axis, but when the true 2D shapes are taken into account, Model C is clearly better than the others.

In summary, we derived a model that is able to fit accurately mapped wild-type data. We next had to seek other experimental support for our specific crossover model. Could our optimized model explain non-wild-type results—the effects of various types of experimental perturbations? If not, the experimental perturbations should be valuable extra constraints with which to improve the model.

### Perturbation experiments I: beads of RA

**Figure 5:**
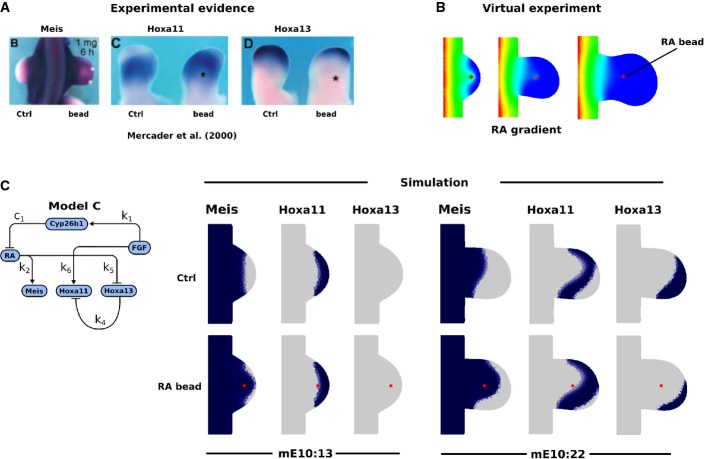
Computational experiments with RA beads Experimental evidence on the effect of applying RA-soaked beads in chick limbs and monitoring the expression patterns of Meis, Hoxa11 and Hoxa13. Data from Mercader *et al* (2000).

A classical type of manipulation used in limb development is the implantation of beads soaked in diffusible proteins or drugs into the limb bud tissue. A previous study on PD patterning (Mercader *et al*, 2000) showed that when RA-soaked beads were implanted in the distal region of chick limb buds, they promote the expression of Meis1 and Meis2 and shifted the expression pattern of Hoxa11–Hoxa13 distally (Fig5A).

A virtual bead moving with the tissue, secretes RA, as a source which decreases exponentially over time.

Simulated expression patterns of Meis, Hoxa11 and Hoxa13 at mE10:13 and mE10:22 for the unperturbed system and for the perturbed system with an implanted RA bead. In the experimental case, the distal boundaries of Meis and Hoxa11 shift distally, while the proximal boundary of Hoxa11 is unaltered. We use here the limb-specific staging system developed in Boehm *et al* (2011), which provides an hour-by-hour temporal resolution (mE10:13 = 10 days and 13 h).

To test our best model from the reverse-engineering process (the crossover model), we reproduced this experiment by simulating a gradually decreasing point source of RA that was carried along with the tissue during outgrowth (Fig5B). A single triangle of the mesh describing the early limb bud was marked as the morphogen source. This point source produced RA at a rate that decreased exponentially over time, simulating the exhaustion of the morphogen. In (15), we show the modified source term for RA in the bead triangle.

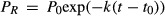
15where tb is the time-point when the bead is placed into the limb. Since the dynamics of RA release from the bead are unknown, we performed virtual bead experiment by testing a variety of different values for the initial RA concentration within the bead (*P*_0_). The results showed that when the exogenous RA is strong enough (*P*_0_ = 20), the expression of the PD markers changes in accordance with the real experiments (Fig5C). In particular, the Meis boundary and the Hoxa11/Hoxa13 boundary shift distally, while, importantly, the proximal Hoxa11 boundary remains unaltered. This result supports the crossover model as it shows that the two genes under the control of RA are shifted, while the boundary under control of FGF (the Hoxa11 proximal boundary) remains unchanged.

### Perturbation experiments II: removal of FGF signals

**Figure 6:**
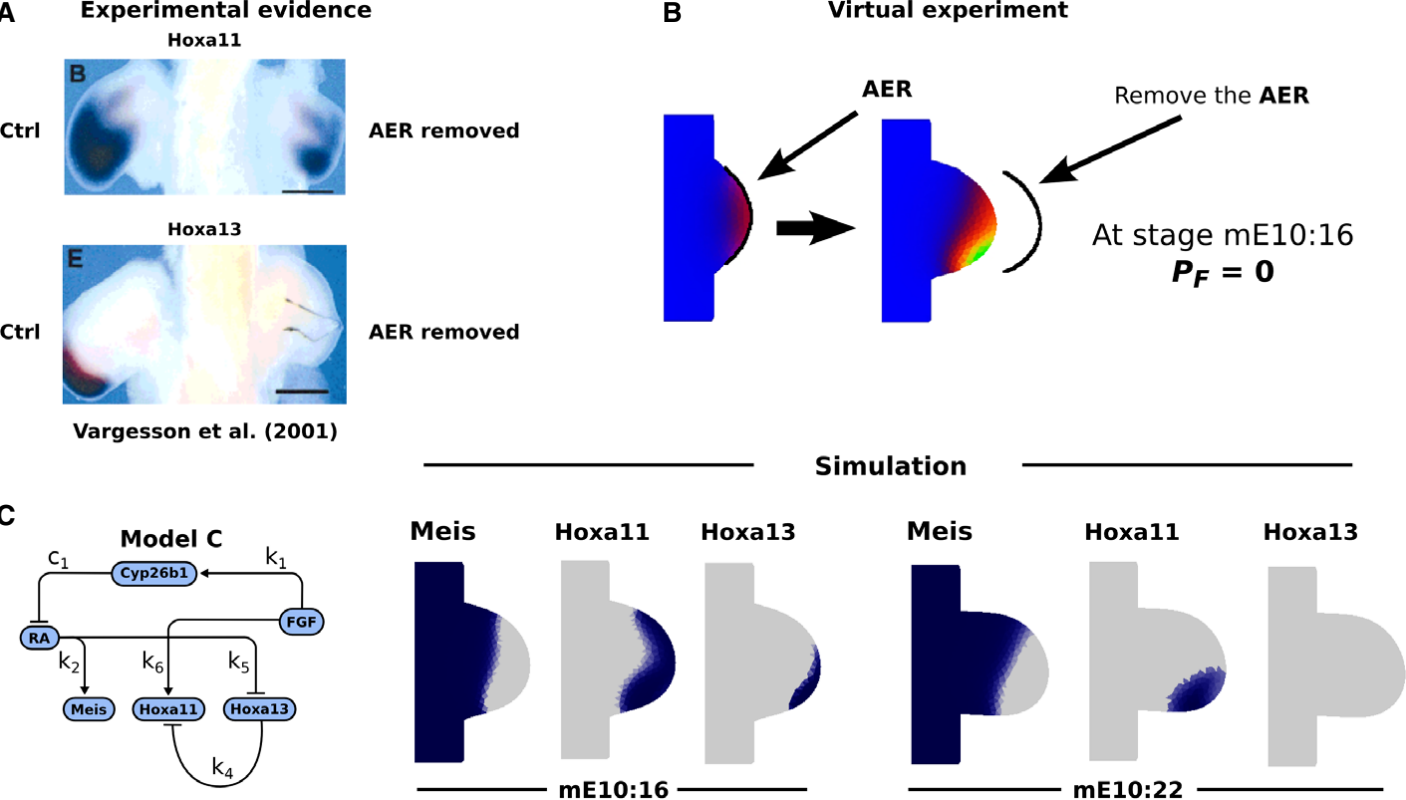
AER removal experiments When the AER is removed from stage 21/22 chick limb buds, in which Hoxa13 was already expressed, Hoxa13 becomes downregulated, while Hoxa11 remains expressed. Experimental data from Vargesson *et al* (2001).

Another study (Vargesson *et al*, 2001) manipulated FGF, the other important morphogen gradient involved in PD patterning. In this study, the AER was removed from chick limb buds at stage 21/22, when Hoxa13 has just been activated. Although the expression of Hoxa13 was lost within a few hours, Hoxa11 was maintained for a longer period of time (Fig6A).

In our virtual experiment, the limb bud grows normally until stage mE10:16 at which point the FGF production is set to 0.

Simulation results using a modified Model C show a rapid Hoxa13 downregulation despite the absence of direct activation by FGF. In the model, this is due to diffusion of RA into the distal tissue.

We reproduced this experiment computationally (using Model C), by terminating the production of FGF (*P*_*F*_ = 0) at stage mE10:16 and measuring the effect 6 h later (Fig6B). Only one parameter from our previous Model C had to be altered for it to correctly reproduce the differential downregulation of Hoxa13 compared to Hoxa11 (Fig6C): the decay rate for Hoxa11 had to be reduced by 60%. The downregulation of Hoxa13 occurred despite the absence of a direct regulatory link from FGF, because in our model when FGF is removed, the absence of Cyp26b1 allows RA to diffuse back into the distal tip.

### Perturbation experiments III: ectopic Meis expression

**Figure 7:**
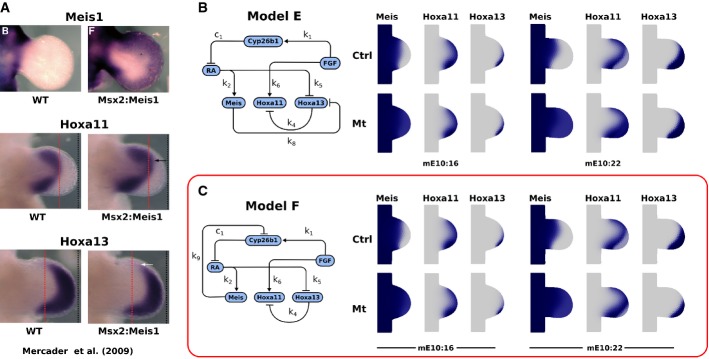
Meis overexpression shifts the Hoxa11–Hoxa13 boundary distally Comparison of the expression patterns of Meis, Hoxa11 and Hoxa13 between the wild-type and Msx2:Meis1 mouse embryos in which Meis1 is overexpressed throughout the limb bud. Experimental data reproduced with permission from Mercader *et al* (2009).

Similar to the RA bead experiments, other manipulative studies (Capdevila *et al*, 1999; Mercader *et al*, 1999; Mercader *et al*, 2009) showed that when Meis1 was misexpressed, the expression of Hoxa11 and Hoxa13 showed a small but reproducible distal shift (Fig7A). Intriguingly as in the RA bead case, although the Hoxa13 expression domain is reduced and the Hoxa11 domain expands distally, there was no clear shift of the proximal Hoxa11 boundary. In our current best model (Model C), Meis has no regulatory effect on any other genes. We therefore tested whether extending the model with a new regulatory link from Meis to the Hox genes could replicate this experimental result. Since ectopic Meis causes shrinkage of the Hoxa13 domain, a simple possibility is that Meis directly represses Hoxa13 (Model E). We implemented this interaction by defining equation 16

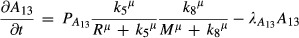
16

In the regulatory network Model E, Meis directly represses Hoxa13; however, this does not result in the correct change in expression patterns (shown at stages mE10:16 and mE10:22).

In the regulatory network Model F, Meis represses Hoxa13 indirectly through Cyp26b1 repression. This is able to reproduce the observed changes in Hox expression patterns.

With normal Meis levels (control in Fig7B), the addition of this new regulatory link had no impact on the gene expression patterns. When we forced Meis expression to occur uniformly throughout the tissue, by adding a constant term to the production rate (equal to *P*_*M*_), the expression patterns of Hox genes changed but they were not in agreement with the patterns of Hoxa11 and Hoxa13 observed in the experiments (Mercader *et al*, 1999). The Hoxa13 expression levels were reduced but its domain shape was unaltered, and Hoxa11 now extended distally until the tip, completely overlapping with Hoxa13. These patterns can be explained by considering the fact that the ectopic expression of Meis was uniform, causing a uniform repression of Hoxa13. Thus, although Hoxa13 expression was lower, its spatial pattern was unaffected, and this in turn had an impact on Hoxa11 which was derepressed throughout the distal Hoxa13 domain.

A particular feature shown by this model was that there was no PD shifting of the original expression boundaries. Indeed, shifting a boundary usually indicates the involvement of a spatial gradient—when the magnitude of the gradient increases or decreases, threshold positions shift spatially. In contrast, the new interaction in Model E links two genes which do not display clear spatial gradients (Meis and Hoxa13). We did not consider a model in which Meis activates the production of RA because there is no RA production in the Meis domain of the wild-type limb bud. We therefore instead explored the possibility that Meis regulates Cyp26b1 because this directly influences the spatial gradient of RA. In particular, we considered that Meis might repress Cyp26b1 (Model F), since it has a positive effect on Hoxa13 (indirectly via repression of RA). This interaction is represented by equation 17.

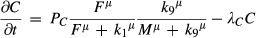
17

Figure7C shows that Model F is indeed able to replicate the experimental results: the indirect repression of Hoxa13 via Cyp26b1 and RA shifts the distal boundary of Hoxa11 distally together with the Hoxa13 boundary, while the proximal boundary of Hoxa11 is unaffected. This is achieved because the proximal boundary is controlled by FGF, which is unaffected by the new link. This again provides very strong support for the crossover model, in which Hoxa13 is primarily controlled by RA, while Hoxa11 is primarily controlled by FGF. As a final check, we retested the new Model F against the previous tests performed in the study. For example, we reran the simulations for the wild-type scenario, the RA-soaked bead experiment and the surgical removal of the AER, and confirmed that the model still gives the same consistent results for all three tests (Supplementary Fig S5). We also performed a sensitivity analysis for each of the free parameters of Model F, and discussed why one of these parameters is less tightly determined than the rest (Supplementary Fig S6).

## Discussion

Reverse-engineering methods use parameter optimization to derive models which can best explain the behaviour of a system. In systems biology, this approach is becoming increasingly important to derive the gene regulatory networks that underlie the temporal dynamics of gene expression and protein distributions. However, this method has so far been limited to cases where the growth of the biological systems could be neglected (e.g. single cells or to static multicellular domains) (Basso *et al*, 2005; Bansal *et al*, 2007; Goutsias and Lee, 2007; Crombach *et al*, 2012). This study provides an example of a reverse-engineering approach applied to an arbitrary growing tissue. It does not change the basic principle of the approach, but it is more computationally challenging and since most developmental pattering occurs in growing tissue, this study extends the method to a greater range of possible model systems. We achieved this result by combining a previously generated model of limb growth (Marcon *et al*, 2011) with gene expression time course data to reverse-engineer the gene regulatory network that controls the PD patterning of the limb.

Although limb PD patterning has traditionally been abstracted as a one-dimensional problem, in this study we have reverse-engineered a two-dimensional model to reproduce the dynamics of the three PD markers. In this way, we were able to use the 2D expression patterns as extra constraints to better distinguish between different hypothesis in the model-fitting process. In particular, we have been able to identify the crossover model as the minimal gene regulatory network that best recapitulates the qualitative shapes of the expression patterns of Meis, Hoxa11 and Hoxa13. We found that the shape of the Hoxa11 pattern could be explained only by a model where RA controlled its distal boundary and FGF controlled its proximal boundary. Importantly, when we extended and analysed the model to reproduce published experimental perturbations, we found further confirmation of the ideas underlying the crossover model. Indeed, in agreement with the experiments, when RA was overexpressed, only the distal boundary of Hoxa11 changed while the proximal boundary was unaffected (Figs5 and 6). This highlights the value of using two-dimensional expression patterns to infer patterning models, since the crossover model, which was derived only by using wild-type expression data, could also reproduce the effect of previously published perturbation experiments.

By analysing other perturbation experiments, we were also able to further extend the crossover model and identify an additional interaction to account for the shift of Hoxa11 and Hoxa13 observed upon Meis overexpression. In particular, we found that the repression of Cyp26b1 by Meis was the simplest additional interaction that could explain this phenomenon. Interestingly, a recent study (Roselló-Díez *et al*, 2014) has analysed the expression of Cyp26b1 in Meis1 misexpression experiments and has arrived to the same conclusion. This further confirms the value of fitting 2D patterns to reverse gene regulatory networks that control patterning. It should be noted that our crossover model depends on a diffusible signal emanating from a straight source in the main body, but it gives no direct evidence about the identity of this molecule, which is still an unresolved question (Cunningham and Duester, 2015).

### Which classical model is correct?

The specification of the main limb axis is a classical model system to study how cells acquire different fates during development. Diffusible morphogens are known to be at the heart of this process; however, the mechanisms of morphogen interpretation are still unclear (Tabin and Wolpert, 2007). In particular, it is still debated whether cells are able to acquire different fates by measuring the relative strengths of morphogen signals, or instead by measuring the duration of exposure to signals. Both ideas have been proposed to explain limb PD patterning. The progress zone model (Summerbell 1974) is a long-standing proposal in support of the signal duration measurement hypothesis. By contrast, the two-signal model (Mercader *et al*, 2000) supports the hypothesis that morphogen signal ratios underlie PD patterning. Finally, a recent study (Roselló-Díez *et al*, 2014) has again revived the idea of a timing mechanism.

**Figure 8:**
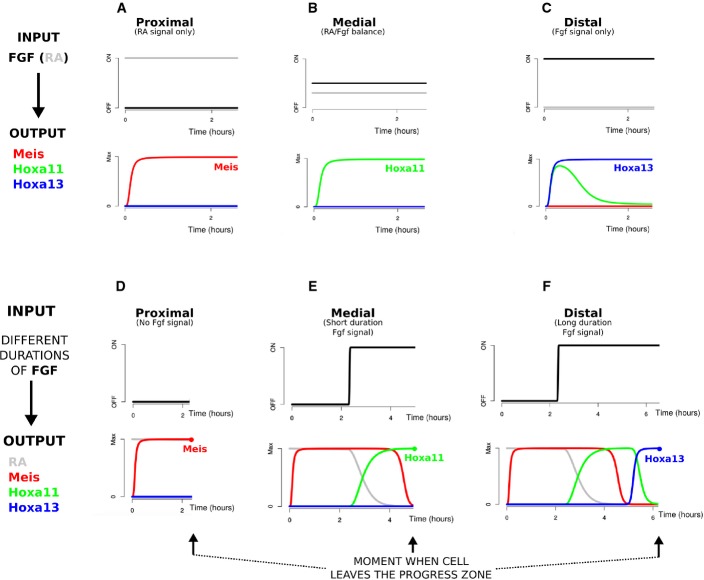
Our regulatory network can be seen both as a two-signal model and a progress zone model. A–C Our regulatory network can describe PD patterning as a balance of two signals. For a proximal cell with high RA and no FGF (A), the stylopod marker Meis is expressed. For a medial cell where both FGF and RA signals are present (B), the zeugopod marker Hoxa11 is stably expressed. For a distal cell with high FGF and no RA (C), the autopod marker Hoxa13 is expressed.

We thus chose to assess which of the two classical hypotheses is a more accurate description of our final model (Model F). In other words, which conceptual behaviours does the model exhibit: levels-measuring or time-measuring? Although our model has been developed in a fully dynamic 2D tissue domain, it can also be used to explore in which way an individual cell could interpret the RA and FGF morphogens to express the different PD markers. To do this, we simply evaluate a single set of the equations without diffusion—equivalent to simulating a single mesh triangle on its own with no neighbours. We first explored the levels-measuring hypothesis and performed simulations by exposing individual cells to different levels of signals for a long period of time. In this scenario, the stable equilibrium would be reached so that the final expression of PD markers would not reflect the temporal integration of the signals but only the signalling ratio of FGF and RA. The plots in Fig8A–C show different temporal dynamics of the input signals and the corresponding PD marker expression levels. These results revealed that Model F was indeed able to measure the relative signalling ratio between RA and FGF: when RA was high and FGF was low, the circuit stably activated Meis (stylopod); when RA and FGF were both at intermediate levels, it promoted Hoxa11 (zeugopod); and when RA was low and FGF was high, it promoted Hoxa13 (autopod).

D–F Our regulatory network can also describe PD patterning as a progress zone model. When there is no FGF signal (D), Meis is expressed. When the FGF signal duration is short (E), Hoxa11 is expressed. And when the FGF signal duration is long (F), Hoxa13 is expressed.

In agreement with the two-signal model, these results showed that our model was able to activate different genes as a function of the signalling ratio between FGF and RA. Nevertheless, we also wanted to test whether the model could measure signal duration. Similar to the previous case, we defined a simulation scenario which could be used to test this hypothesis. We performed simulations by using different durations of FGF signalling but keeping the FGF level at a constant fixed value. In other words, we assumed that the FGF signal could only have one level when it was ON (high value), and would otherwise be OFF (zero value), and we varied only the duration for which FGF was ON. This was not intended as a realistic representation of FGF levels in the real limb bud (which clearly display graded signalling), but it was necessary to perform a clean test of how the network could respond to different durations of FGF signalling. Interestingly, our analysis (Fig8D–F) revealed that the network (with the same parameter values as before) could switch on different genes in response to different durations of FGF exposure and therefore could also function as a temporal integrator of FGF over time. Indeed, in agreement with the progress zone model, when FGF was ON for 0 h, Meis was expressed; when FGF was ON for an intermediate exposure time, Hoxa11 was expressed; and finally, if a cell was exposed to FGF for longer time, it expressed Hoxa13. The precise durations of FGF exposure required for medial versus distal fate will depend on the detailed parameter values—this simulation is simply a proof of concept. Nevertheless, our analysis revealed that a gene regulatory network can operate both as a levels-measuring circuit and as a time-measuring circuit, showing that these two mechanistic concepts may not be easily separable in real systems. This is evident even in the scenario where the signalling ratio is measured (Fig8A–C): it takes some time before the equilibrium is reached, so the early response of the circuit will always exhibit a degree of temporal integration. This highlights a more general point about gene regulatory networks. Since they are intrinsically dynamical systems, some degree of temporal integration will be inevitable, and it may be a general fallacy to consider levels-measuring versus time-measuring as alternative explanations of a given system. In support of this, a very similar observation has also been made in the case of *Sonic hedgehog* signalling in the neural tube, where it is clear that both signal level and signal duration affect the downstream gene expression (Balaskas *et al*, 2012). In the case of limb development, we believe this understanding may underlie the historical difficulties in resolving the debate between the PZM and 2SM hypothesis. It reveals that a single gene regulatory network underlying PD patterning could simultaneously be explained by the two different conceptual models.

## Materials and Methods

### Mapping gene expression data

We included in our limb bud growth model experimental data with the expression of different genes. Gene expression data were obtained using whole-mount *in situ* hybridization techniques from mouse forelimbs and hindlimbs at different stages of development. Given an image corresponding to the expression of a gene at some stage in development, we used a combination of techniques to map it to our 2D limb bud growth model (Marcon *et al*, 2011) and convert it to molecular concentrations. First, using a limb bud morphometric tool, we obtained the precise stage of the limb bud comparing its shape to a database of limb bud shapes from different stages of development (Boehm *et al*, 2011). Then, the image was processed and edited to remove the background noise and obtain the expression pattern. Using a computational tool developed in the laboratory, we aligned the processed image to the limb bud model at the correct stage and converted light intensity values to molecular concentrations in such a way that highest intensity values corresponded to a concentration of 1 and lowest values to a concentration of 0. We repeated the same process for the different stages of development for which we have experimental data. We obtained interpolated gene expression patterns for intermediate time-points using the tissue movement map implemented in the model.

### Optimizing a regulatory model

For each network model (each topology), we used parameter optimization techniques to find the best parameter set that fitted the available experimental data. We used a gradient descent algorithm implemented in the Root package (Brun and Rademakers, 1997) (https://root.cern.ch). During the optimization process, some of the parameters were left free, while others were fixed (see main text). In particular, *P*_*FGF*4_, *λ*_*F*_, *λ*_*R*_, *μ*, 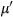, *c*_1_, *k*_1_, *k*_2_, *k*_3_, *k*_4_, *k*_5_, *k*_6_ and *k*_7_ were free to optimize, while *D*_*F*_, *D*_*R*_, *P*_*FGF*8_, *P*_*R*_, *P*_*C*_, *P*_*M*_, 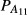, 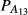, *λ*_*C*_, *λ*_*M*_, 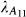 and 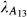 were fixed. All values of parameters used in the simulations are detailed in Supplementary Table S2. As experimental data, we used the WT gene expression patterns of Meis, Hoxa11 and Hoxa13 at different stages of development (mE10:9 to mE11:12) obtained from mouse forelimbs and hindlimbs using *in situ* hybridization techniques. We used 4, 26 and 32 different time-points for Meis, Hoxa11 and Hoxa13, respectively (more time-points for the patterns with more complex or dynamic shapes). To each simulation, we assigned a score defined as a weighted sum of square differences between experimental and simulated concentrations of Meis, Hoxa11 and Hoxa13 at different stages of development. We applied a non-linear scaling to the experimental and simulated data so that the emphasis was on the shapes of the gene expression patterns rer than the levels. We used a scaling Hill function given by 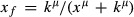, where *x* is the non-filtered data, *x*_*f*_ is the filtered data, and *k* and *μ* are Hill parameters. In Supplementary Fig S3, we show that the choice of best model is not affected by the parameter values chosen. The weighting for Hoxa11 at the last time-point was assigned to 20 because its more complex pattern is particularly important to recreate. We optimized the parameters of each tested model a total of 27 times, each time starting from a different initial parameter set (see Supplementary Fig S1). To have a sufficiently diverse initial parameter set, we chose three upstream parameters that were affecting all the genes *λ*_*F*_, *λ*_*R*_ and *c*_1_ and for each one selected three reasonable values spanning two orders of magnitude (values given in Supplementary Table S1). Combining these three parameter values gave 27 combinations, and the remaining free parameters were chosen at random (see Supplementary Table S1, and the convergence of these values in Fig4J–L).

Our model starts at a time-point when the RA and FGF gradients already exist. We therefore “presimulated” the diffusion of these gradients for the equivalent of 3 h on the static mesh representing the first shape of the model—mE10:09. The remaining molecules were all set to a concentration of zero at the beginning of the simulations.

## Supporting Information

**Supplementary Information**

**Review Process File**
