# Supplementary material for: Data-driven modelling of a gene regulatory network for cell fate decisions in the growing limb bud
Source: Mol Syst Biol. 2015 Jul 14;11(7):815. doi: 10.15252/msb.20145882 (PMC4547844; doi:10.15252/msb.20145882)
Supplement: Supplementary file 1 [file msb0011-0815-sd1.pdf]

Data-driven modelling of a gene regulatory network for cell fate decisions in the growing limb bud.
Supplementary Information

List of Figures

1 Exploration of parameter space. 2
2 Initial parameter values for the optimisations. 3
3 Non-linear rescaling of expression data does not alter the optimisation results. 4
4 Effect of removing almost half the timepoints of the experimental data. 4
5 Exploration of non-minimal models supports Model C. 5
6 Re-testing Model F against all experimental evidence. 6
7 Sensitivity analysis. 7
8 Parameter values for all shown models. 8
9 Raw data 11

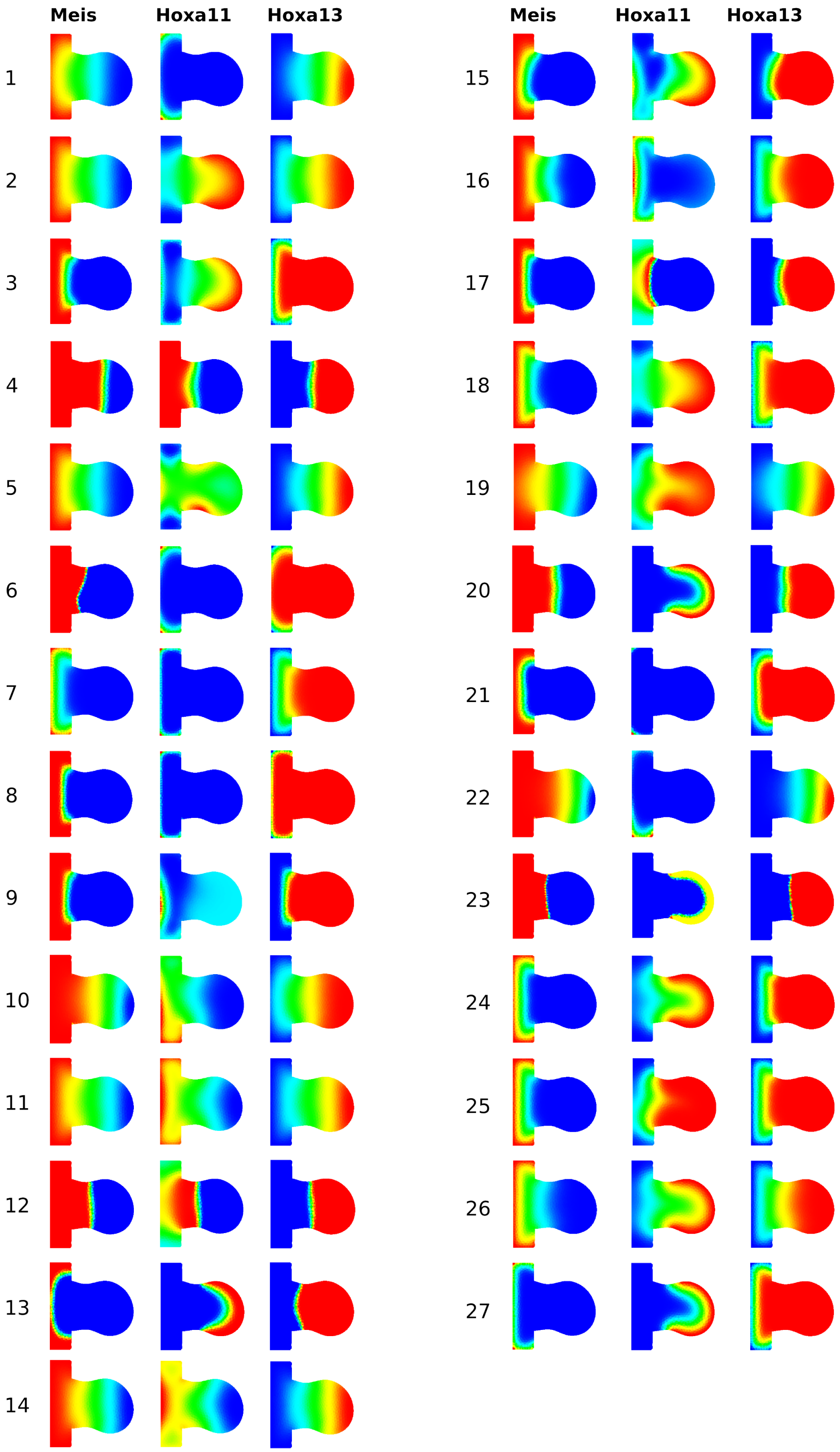

Supplementary Figure S1: Exploration of parameter space. Simulations of the 27 initial parameter combinations for Model C, show the variety of the starting conditions for the optimisations, and also how different they are from the target pattern (the experimental data).

Index	P_{FGF4}	μ	μ'	λ_F	λ_R	c_1	k_1	k_2	k_3	k_4	k_5	k_6	k_7
1	3.15E-01	3.89E-01	9.69E+00	1.00E-04	1.00E-03	1.00E-02	3.72E-04	2.59E-01	4.29E-03	3.81E-01	3.40E-05	5.45E-05	4.21E-04
2	5.96E-04	1.71E-01	2.05E-01	1.00E-04	1.00E-03	1.00E-01	2.65E-01	4.77E-03	4.24E-01	4.10E-01	1.54E-05	7.91E-03	5.63E-01
3	7.39E-02	6.55E-01	1.63E+00	1.00E-04	1.00E-03	1.00E+00	1.22E-01	5.49E-05	4.14E-05	6.48E-01	2.76E-05	1.30E-02	8.85E-02
4	5.24E-01	4.17E+00	4.65E-01	1.00E-04	1.00E-02	1.00E-02	2.25E-02	8.40E-04	2.12E-02	1.07E-04	7.41E-02	8.60E-02	4.04E-03
5	4.35E-02	2.74E-01	1.95E-01	1.00E-04	1.00E-02	1.00E-01	3.32E-01	3.23E-03	2.15E-05	2.69E-03	1.06E-05	2.88E-02	5.33E-05
6	9.60E-01	3.27E+00	6.52E-01	1.00E-04	1.00E-02	1.00E+00	4.10E-01	1.74E-04	6.73E-02	3.14E-05	2.45E-03	9.11E-03	9.72E-01
7	3.43E-03	5.24E-01	5.92E+00	1.00E-04	1.00E-01	1.00E-02	4.16E-01	4.88E-01	2.06E-03	4.72E-04	3.66E-04	4.07E-04	1.62E-02
8	1.68E-01	2.27E+00	2.13E+00	1.00E-04	1.00E-01	1.00E-01	1.16E-01	2.83E-03	1.77E-03	3.72E-04	1.19E-05	4.08E-03	2.70E-01
9	5.87E-01	1.58E+00	6.05E-01	1.00E-04	1.00E-01	1.00E+00	8.69E-01	8.35E-04	1.76E-05	2.00E-01	1.74E-02	7.82E-04	1.63E-03
10	4.43E-01	5.73E-01	5.70E-01	1.00E-03	1.00E-03	1.00E-02	7.92E-05	5.72E-05	2.53E-04	1.05E-03	8.55E-02	4.95E-03	1.67E-01
11	9.30E-04	1.12E-01	5.54E-01	1.00E-03	1.00E-03	1.00E-01	6.31E-02	1.82E-05	4.18E-04	7.06E-05	7.78E-03	1.44E-03	3.34E-04
12	5.13E-03	1.67E+00	9.91E-01	1.00E-03	1.00E-03	1.00E+00	1.02E-01	7.91E-05	9.17E-04	2.81E-03	1.26E-04	2.82E-01	1.25E-04
13	4.93E-03	8.94E+00	1.03E-01	1.00E-03	1.00E-02	1.00E-02	3.65E-03	2.25E-01	4.76E-05	1.13E-01	2.75E-02	4.13E-04	1.77E-02
14	2.37E-03	2.54E-01	6.01E-01	1.00E-03	1.00E-02	1.00E-01	3.91E-02	1.66E-05	3.03E-05	3.89E-04	2.57E-03	5.99E-01	2.30E-05
15	5.43E-01	8.34E-01	5.25E-01	1.00E-03	1.00E-02	1.00E+00	4.71E-01	7.32E-03	3.50E-05	3.59E-01	3.06E-04	5.92E-05	1.03E-03
16	5.12E-04	4.03E-01	9.51E-01	1.00E-03	1.00E-01	1.00E-02	9.83E-05	7.58E-05	2.69E-05	4.28E-02	5.22E-03	5.80E-05	2.98E-03
17	8.91E-04	1.35E+00	3.61E+00	1.00E-03	1.00E-01	1.00E-01	3.22E-02	2.70E-03	2.12E-02	9.11E-03	4.56E-03	2.12E-02	3.55E-05
18	1.98E-03	2.84E-01	1.10E-01	1.00E-03	1.00E-01	1.00E+00	1.21E-01	6.98E-05	1.07E-01	3.14E-04	7.93E-05	3.52E-04	8.24E-01
19	3.98E-01	3.23E-01	2.14E-01	1.00E-02	1.00E-03	1.00E-02	6.80E-01	5.53E-04	2.78E-01	3.64E-05	2.99E-01	4.28E-03	1.52E-03
20	3.83E-02	1.39E+00	4.13E-01	1.00E-02	1.00E-03	1.00E-01	2.64E-04	8.71E-04	9.13E-05	1.40E-02	1.16E-02	1.03E-02	1.33E-02
21	6.41E-04	8.72E-01	7.63E+00	1.00E-02	1.00E-03	1.00E+00	2.35E-05	3.49E-03	3.34E-02	1.60E-01	4.79E-05	1.04E-04	3.34E-02
22	4.57E-01	7.01E-01	9.20E+00	1.00E-02	1.00E-02	1.00E-02	1.96E-01	7.78E-05	8.24E-04	5.12E-02	5.05E-03	7.27E-03	7.24E-04
23	1.07E-03	8.27E+00	1.38E-01	1.00E-02	1.00E-02	1.00E-01	4.31E-05	5.88E-04	4.12E-03	1.14E-02	1.73E-02	5.63E-01	1.37E-04
24	4.27E-03	4.00E-01	4.93E-01	1.00E-02	1.00E-02	1.00E+00	4.43E-05	8.01E-03	1.01E-04	5.80E-04	1.30E-01	4.24E-01	6.94E-05
25	2.67E-01	7.04E-01	1.20E-01	1.00E-02	1.00E-01	1.00E-02	3.53E-04	1.20E-02	2.25E-04	6.35E-05	9.38E-03	1.34E-04	1.55E-02
26	5.02E-02	2.34E-01	2.00E-01	1.00E-02	1.00E-01	1.00E-01	1.05E-03	8.47E-02	2.52E-02	5.33E-03	8.43E-03	3.36E-02	5.43E-03
27	2.66E-01	1.13E+00	1.09E+00	1.00E-02	1.00E-01	1.00E+00	3.97E-02	9.29E-01	5.35E-04	4.27E-02	9.17E-02	1.37E-04	6.34E-02

Supplementary Table S1: Initial parameter values for the optimisations. Each parameter set corresponds to the images of the same number in Supplementary Figure S1.

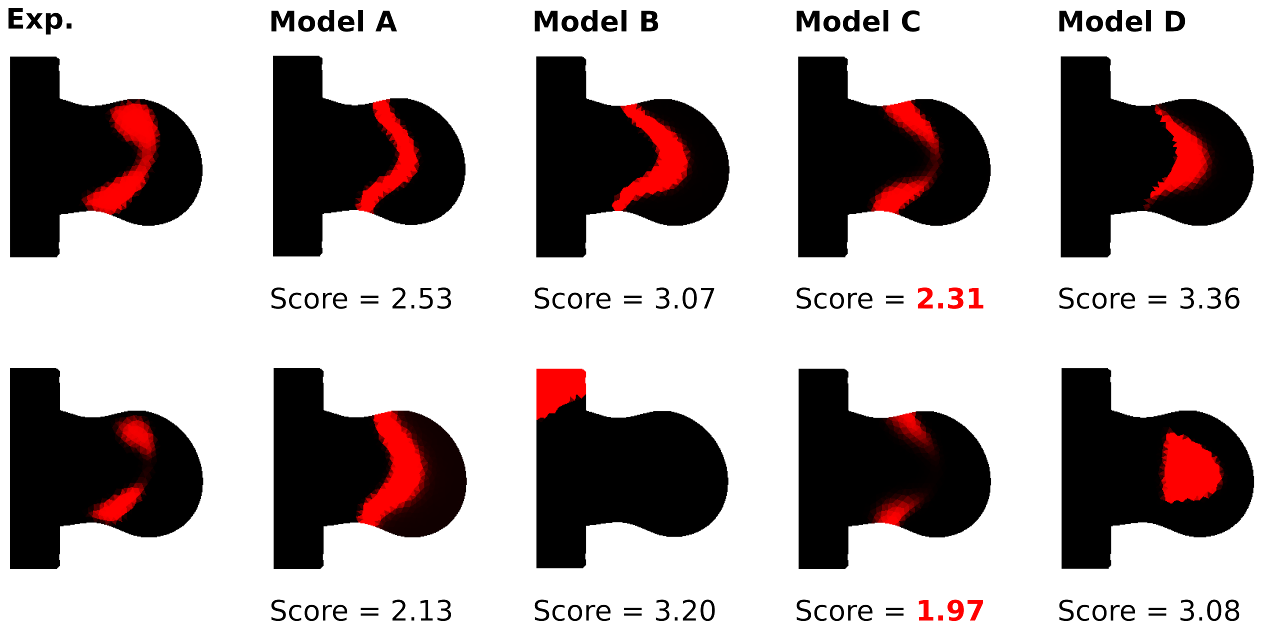

Supplementary Figure S2: Non-linear rescaling of expression data does not alter the optimisation results. The top row shows the experimental data plus the best optimisation results for each of the 4 models. In each case the non-linear transform described in the Methods section has been applied. The scores underneath show that Model C provides the best score (ie. the smallest difference from experimental data). The bottom row shows the same analysis, but for a different non-linear scaling of the data, which is equivalent to under-developing the in-situ hybridisation for the experimental data (first column). Although the absolute scores change, the best model overall is again Model C.

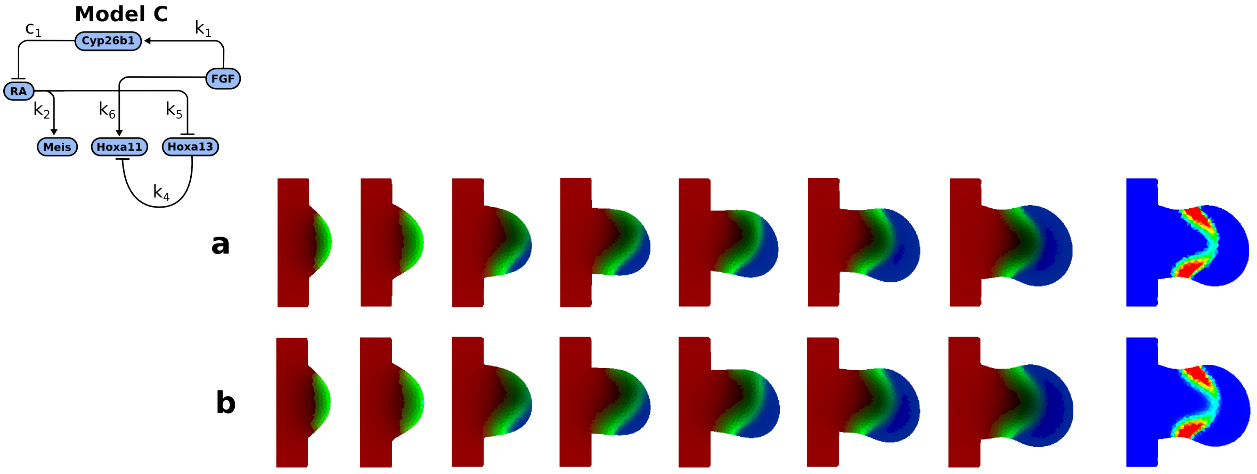

Supplementary Figure S3: Effect of removing almost half the timepoints of the experimental data. When every second timepoint of mapped expression data was removed from the fitting procedure for Hoxa11 and Hoxa13, the optimised Model C (a) was still visually indistinguishable from the original result (b).

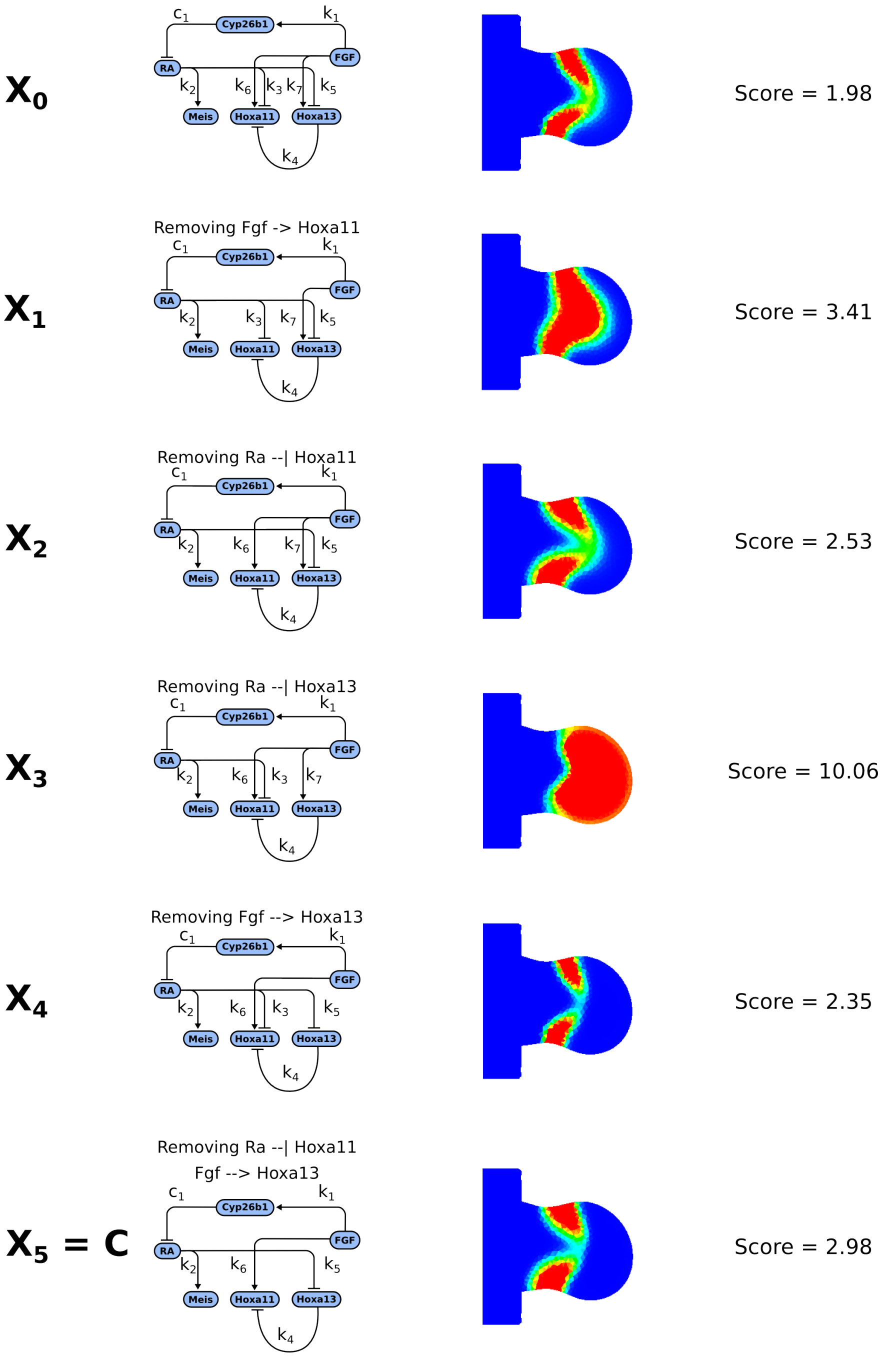

Supplementary Figure S4: Exploration of non-minimal models supports Model C. We chose to optimise another model (Model X_0), which rather than being one of the simplest topologies, represents the "most complex" topology, ie. it contains all the possible regulatory links between the upstream nodes RA/FGF and the downstream nodes Hox genes (see Figure 1e). We optimized this "super-model" X_0 and then tested it by removing each regulatory link one-by-one, thereby testing a series of intermediate models, X_1-X_4. When either the link from FGF to Hoxa11 was removed (X_1), or the link from RA to Hoxa13 (X_3), the resulting pattern (and score) dramatically worsened. By contrast, if the link from RA to Hoxa11 was removed (X_2), or the link from FGF to Hoxa13 (X_4), this had much less impact on the resulting Hoxa11 pattern. In other words, the links which are most important to maintaining a good score are the two links of Model C. Indeed, when we remove both of the unimportant links (X_5), we have recreated Model C and the resulting pattern is almost as good as when Model C was optimised directly.

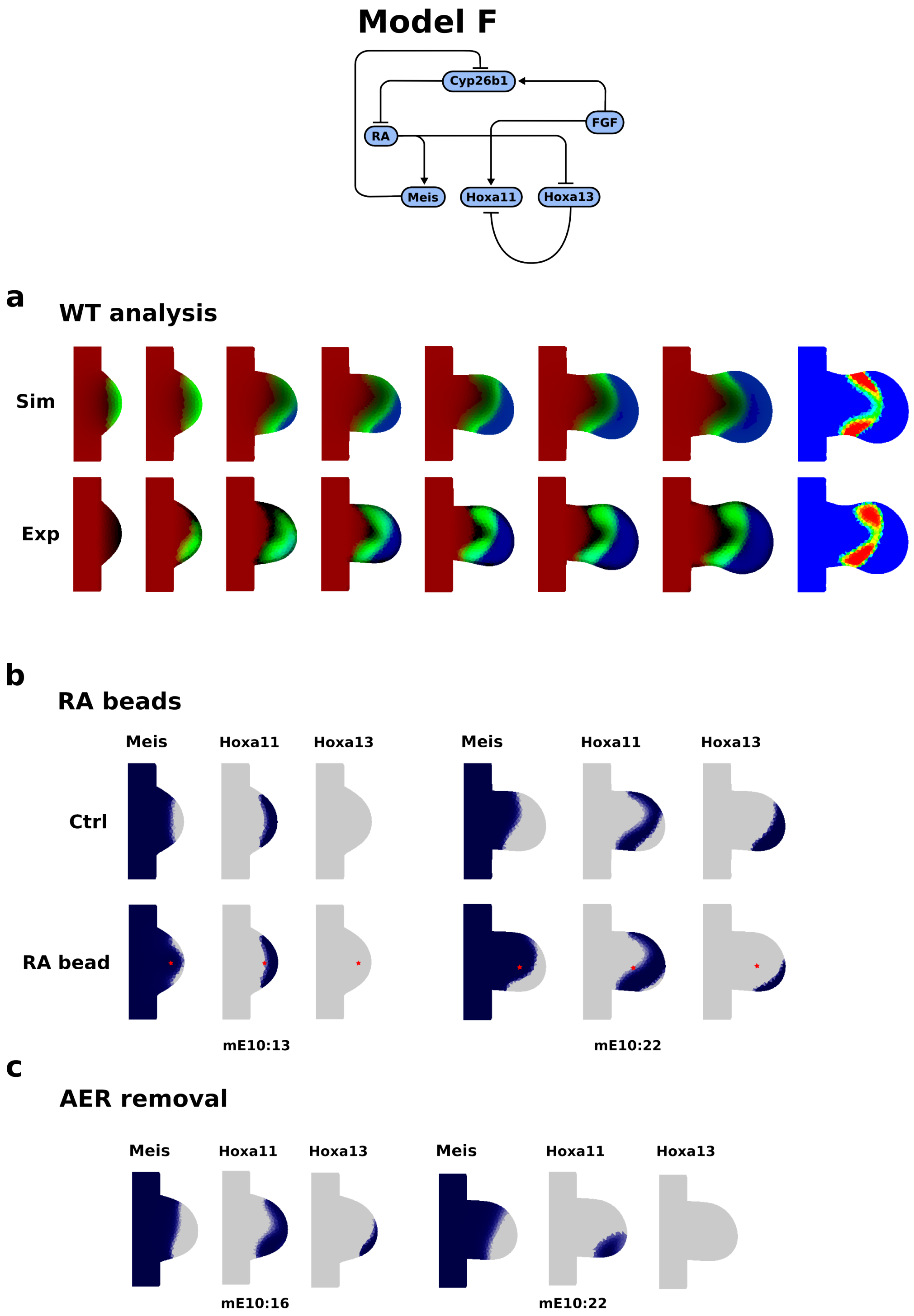

Supplementary Figure S5: Re-testing Model F against all experimental evidence. Model F is derived from Model C, but with 2 changes: a 60% reduced decay rate for Hoxa11, and the addition of a regulatory link (k9) to explain the Meis ectopic expression results. We therefore went back to re-test the final model against: (a) the wildtype time course, (b) the RA-bead experiments, (c) the AER-removal experiments. The results were unchanged from before, which is expected because Hoxa11 does not regulate any other genes in the system, the new k9 link is not activated during any of these experiments, as the expression patterns of Meis and Cyp26b1 do not overlap in any of these cases.

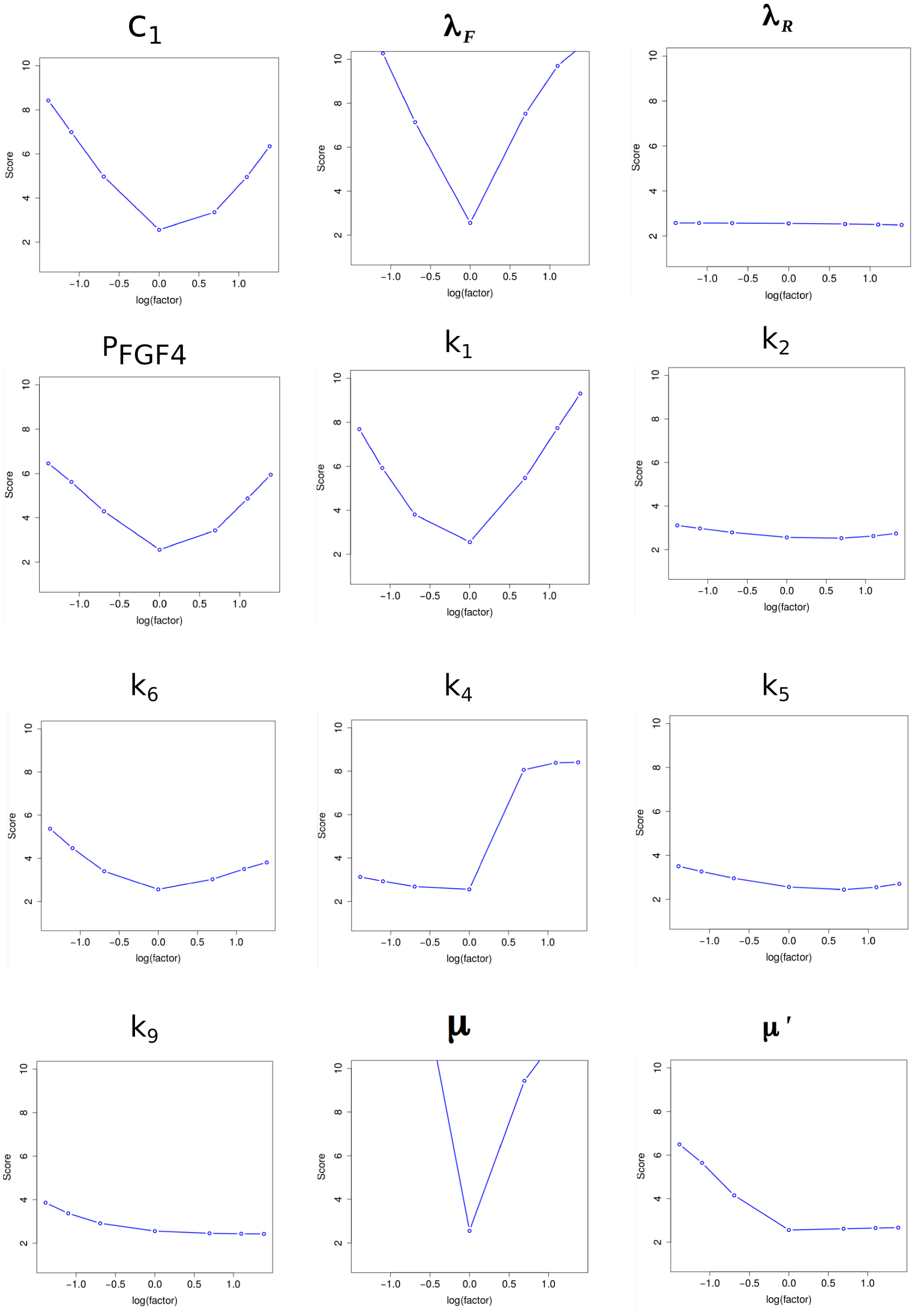

Supplementary Figure S6: Sensitivity analysis. On the final model of our study, Model F, we performed a sensitivity analysis for the free parameters. Each parameter was gradually increased and decreased up to a 4-fold difference in each direction, and the resulting score of the simulated model was assessed. The results show that most parameters are well-determined (considering that even a difference score of 3.0 is a noticeably bad reproduction of the expression patterns, e.g. Figure 3b). Only one parameter is completely insensitive to variations: lambda-R, the background degradation of RA, can take almost any value because in the successful model, RA is strongly regulated by Cyp26b1. It suggests that the model can operate successfully without this degradation term in equation (3), and indeed subsequent tests confirmed that it can be removed.

Supplementary Table S2: Parameter values for all shown models. The parameters in bold are parameters that were optimized automatically while the other parameters were given fixed values. The diffusion constants were taken from the literature (see main text). Most production rates (P) and decay rates (λ) were fixed to 0.05, such that maximal expression would equilibrate to a relative concentration of 1.0. We will comment briefly on the values of the free parameters just for Model C, as this is the successfully optimised one. The final model (F) is directly derived from C with just the addition of the k_9 link. Production rates are relative, and the optimised value for FGF4 is less than an oder of magnitude different from the fixed value for FGF8. Decay rates are very low, but the lower value λ_R, is also very under-determined (see Supplementary Figure S6) and so this value is not important (and can be neglected from the model). The regulatory cooperativity represented by μ and μ' are very reasonable for non-liner biomolecular systems. The k values all appear reasonable, except k_5 which seems very small relative to the others. In fact, for repressive interactions a smaller value represents a stronger repression. In the case of k_5 it is clear that Hoxa13 is very sensitive to even low levels of RA, and this is in agreement with the literature cited in the main text.

Model A

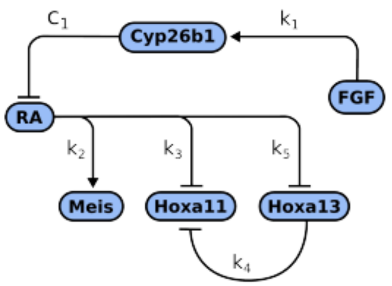

Parameter	Value	Units
D_F	100	μm^2*min^{-1}
D_R	600	μm^2*min^{-1}
P_{FGF8}	0.05	min-1
P_{FGF4}	**4.99**	min-1
P_R	1	min-1
P_C	0.05	min-1
P_M	0.05	min-1
P_{A11}	0.05	min-1
P_{A13}	0.05	min-1
λ_F	**0.0368**	min^{-1}
λ_R	**0.000103**	min^{-1}
λ_C	0.05	min^{-1}
λ_M	0.05	min^{-1}
λ_{A11}	0.05	min^{-1}
λ_{A13}	0.05	min^{-1}
μ	**1.28**	
μ'	**1.56**	
c_1	**0.238**	min-1
k_1	**0.00259**	
k_2	**0.00814**	
k_3	**0.00259**	
k_4	**0.171**	
k_5	**0.00404**	
Best Score = 2.53		

Model B

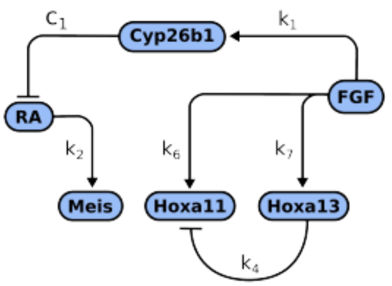

Parameter	Value	Units
D_F	100	μm^{2}*min^{-1}
D_R	600	μm^{2}*min^{-1}
P_{FGF8}	0.05	min-1
P_{FGF4}	**0.141**	min-1
P_R	1	min-1
P_C	0.05	min-1
P_M	0.05	min-1
P_{A11}	0.05	min-1
P_{A13}	0.05	min-1
λ_F	**0.00291**	min^{-1}
λ_R	**0.00441**	min^{-1}
λ_C	0.05	min^{-1}
λ_M	0.05	min^{-1}
λ_{A11}	0.05	min^{-1}
λ_{A13}	0.05	min^{-1}
μ	**5.74**	
μ'	**1.91**	
c_1	**0.0103**	min^{-1}
k_1	**0.0237**	
k_2	**0.130**	
k_4	**0.0477**	
k_6	**0.119**	
k_7	**0.111**	
Best Score = 3.07		

Model C

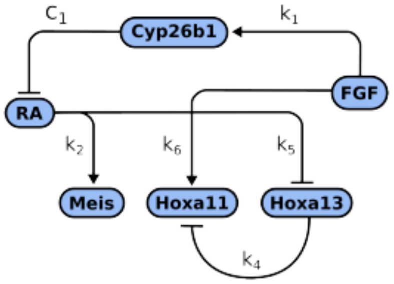

Parameter	Value	Units
D_F	100	μm^2*min^{-1}
D_R	600	μm^2*min^{-1}
P_{FGF8}	0.05	min-1
P_{FGF4}	**0.335**	min-1
P_R	1	min-1
P_C	0.05	min-1
P_M	0.05	min-1
P_{A11}	0.05	min-1
P_{A13}	0.05	min-1
λ_F	**0.00704**	min^{-1}
λ_R	**0.000125**	min^{-1}
λ_C	0.05	min^{-1}
λ_M	0.05	min^{-1}
λ_{A11}	0.05	min^{-1}
λ_{A13}	0.05	min^{-1}
μ	**1.25**	
μ'	**3.77**	
c_1	**2.46**	min^{-1}
k_1	**0.353**	
k_2	**0.145**	
k_4	**0.622**	
k_5	**0.000699**	
k_6	**0.0604**	
**Best Score = 2.41**		

Model D

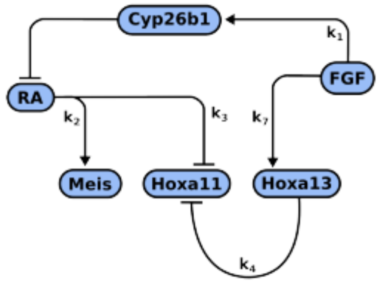

Parameter	Value	Units
D_F	100	μm^2*min^{-1}
D_R	600	μm^2*min^{-1}
P_{FGF8}	0.05	min-1
P_{FGF4}	**0.174**	min-1
P_R	1	min-1
P_C	0.05	min-1
P_M	0.05	min-1
P_{A11}	0.05	min-1
P_{A13}	0.05	min-1
λ_F	**0.00324**	min^{-1}
λ_R	**0.000159**	min^{-1}
λ_C	0.05	min^{-1}
λ_M	0.05	min^{-1}
λ_{A11}	0.05	min^{-1}
λ_{A13}	0.05	min^{-1}
μ	**5.34**	
μ'	**0.575**	
c_1	**0.997**	min^{-1}
k_1	**0.0598**	
k_2	**0.156**	
k_3	**0.0235**	
k_4	**0.0359**	
k_7	**0.115**	
**Best Score = 3.36**		

Model E

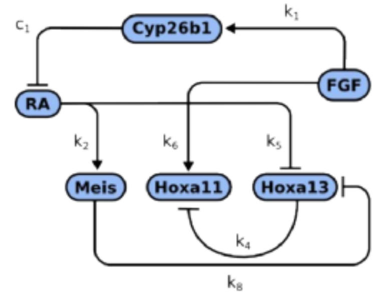

Model F

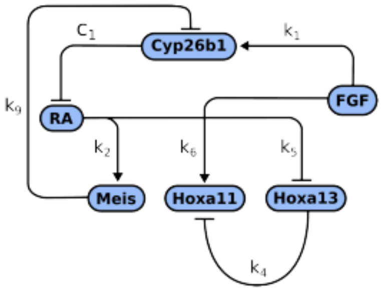

Parameter	Value	Units
D_F	100	$\mu m^2 * min^{-1}$
D_R	600	$\mu m^2 * min^{-1}$
P_{FGF8}	0.05	min-1
P_{FGF4}	**0.335**	min-1
P_R	1	min-1
P_C	0.05	min-1
P_M	0.05	min-1
P_{A11}	0.05	min-1
P_{A13}	0.05	min-1
λ_F	**0.00704**	min^{-1}
λ_R	**0.000125**	min^{-1}
λ_C	0.05	min^{-1}
λ_M	0.05	min^{-1}
λ_{A11}	0.02	min^{-1}
λ_{A13}	0.05	min^{-1}
μ	**1.25**	
μ'	**3.77**	
c_1	**2.46**	min^{-1}
k_1	**0.353**	
k_2	**0.145**	
k_4	**0.622**	
k_5	**0.000699**	
k_6	**0.0604**	
k_8	1.00	
**Best Score = 2.41**		

Parameter	Value	Units
D_F	100	$\mu m^{2} * min^{-1}$
D_R	600	$\mu m^{2} * min^{-1}$
P_{FGF8}	0.05	min-1
P_{FGF4}	**0.335**	min-1
P_R	1	min-1
P_C	0.05	min-1
P_M	0.05	min-1
P_{A11}	0.05	min-1
P_{A13}	0.05	min-1
λ_F	**0.00704**	min^{-1}
λ_R	**0.000125**	min^{-1}
λ_C	0.05	min^{-1}
λ_M	0.05	min^{-1}
λ_{A11}	0.02	min^{-1}
λ_{A13}	0.05	min^{-1}
μ	**1.25**	
μ'	**3.77**	
c_1	**2.46**	min^{-1}
k_1	**0.353**	
k_2	**0.145**	
k_4	**0.622**	
k_5	**0.000699**	
k_6	**0.0604**	
k_9	1.00	
**Best Score = 2.55**		

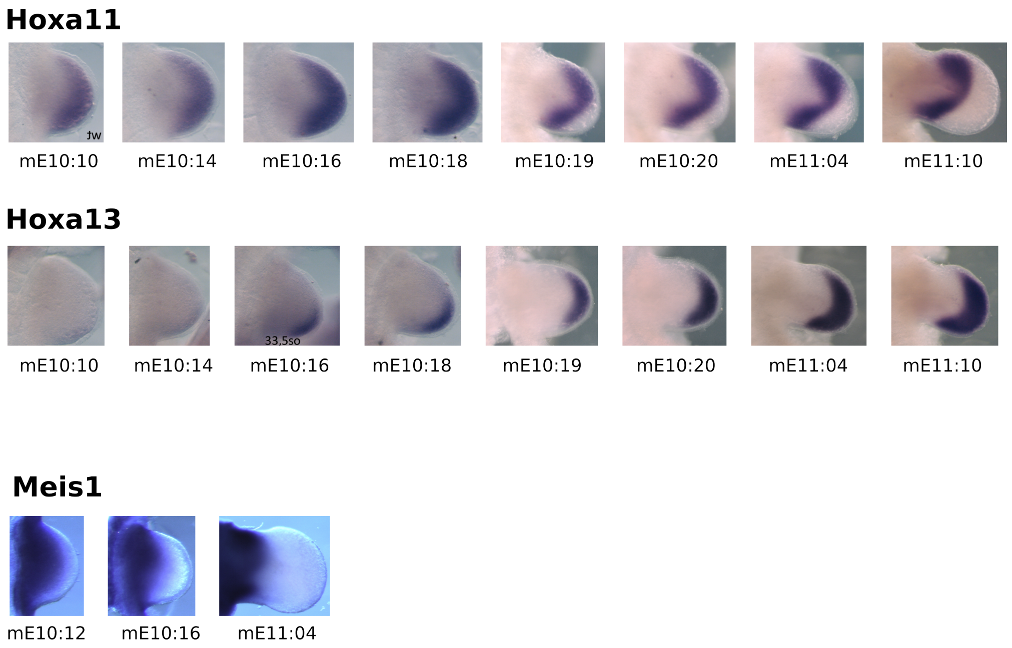

Supplementary Figure S7: Raw data Photos of expression patterns for Hoxa11 and Hoxa13 were kindly provided by Nadia Mercader and Miguel Torres
