# Supplementary material for: Data-driven modelling of a gene regulatory network for cell fate decisions in the growing limb bud
Source: Mol Syst Biol. 2015 Jul 14;11(7):815. doi: 10.15252/msb.20145882 (PMC4547844; doi:10.15252/msb.20145882)
Supplement: Supplementary file 2 [file msb0011-0815-sd2.pdf]

Data-driven modelling of a gene regulatory network for cell fate decisions in growing tissue

Manu Uzkudun, Luciano Marcon, James Sharpe

Corresponding author: James Sharpe, ICREA - Centre for Genomic Regulation

Review timeline:

Submission date:	31 October 2014
Editorial Decision:	15 December 2014
Revision received:	25 March 2015
Pre-acceptance letter:	21 April 2015
Accepted:	11 May 2015

Editor: Maria Polychronidou

Transaction Report:

(Note: With the exception of the correction of typographical or spelling errors that could be a source of ambiguity, letters and reports are not edited. The original formatting of letters and referee reports may not be reflected in this compilation.)

1st Editorial Decision 15 December 2014

Thank you again for submitting your work to Molecular Systems Biology. We have now heard back from the three referees who agreed to evaluate your manuscript. As you will see from the reports below, the referees acknowledge that the presented analyses seem potentially interesting. However, they raise a series of concerns, which should be carefully addressed in a revision of the manuscript.

Without repeating all the comments listed below, some of the more fundamental issues are the following:
- Sensitivity analyses should be performed and parameter choice needs to be better justified.
- As referee #1 points out, further analyses are required to more convincingly demonstrate that the final network fits all the perturbation phenotypes.
- Moreover, the reviewers list a series of technical issues that need to be carefully addressed and they refer to the need to clarify and/or discuss in better detail, several points throughout the manuscript.

Reviewer #1:

Uzkudun et al take a computational approach to test and refine a gene regulatory network that specifies proximodistal (PD) identity in the vertebrate limb. The authors use model optimization approaches, constrained by experimentally measured spatial patterns of gene expression, to identify models that interpret anti-parallel gradients of FGF and RA. The authors benchmark the model they derive against previous experimental perturbations of the tissue. A counter-intuitive observation from the model is that proximal morphogen (RA) appears to control the distal boundary of the target gene HoxA11 whereas the distal morphogen (FGF) controls its proximal boundary.

© European Molecular Biology Organization

Overall this is a well performed study combining experimental and computational approaches. The conclusions are novel and will be of broad interest to developmental and systems biologists. The importance of the analysis is that the mechanism the authors propose reconciles two previous proposed models. These previous models were thought to be contradictory but Uzkudun et al show that an understanding of the dynamics of the gene regulatory network reveals that the two models can be implemented by the same dynamical system. The study nicely demonstrates the power of an interdisciplinary approach to provide new insight and describes the approach in a simple and accessible manner.

Nevertheless I think there are several issues that should be addressed to strengthen and clarify the study.

- The authors describe their approach as a "novel computation approach for reverse engineering gene regulatory networks". This might be a semantic issue and it doesn't detract from the value of the work, but I found this description misleading. First, it seems to imply that a network will be inferred from the data. Instead the authors are using model optimization techniques to parameterize differential equation models that describe the evolution of gene expression on a growing domain. There is no inference of topology and the 'reverse engineering' refers to the use of experimental gene expression patterns to develop target descriptions for the optimization. Second, I'm unclear what is 'novel' about the computational approach. The authors are using a gradient descent algorithm, which although not described in detail or referenced, appears to be a standard package. I would agree with the authors that bringing together the various aspects of the approach is novel, but I don't think this can really be labelled a 'novel computational approach'.
I would suggest the authors use a different form of words to describe the approach in the Title and Abstract, so that it does not lead readers to expect a different type of study. In addition the authors should provide more details of how the model optimization was performed.

- The authors place some emphasis on the idea that their work is the first "automated reverse-engineering... on a 2D growing/moving tissue". This may be the case. However the authors don't make clear why this is particularly challenging. Including growth does not appear to change the distance function(Eqn 6) used for scoring the optimization.

- Related to this the authors explain that the growth simulations involve refining a triangular mesh describing the tissue by adding new nodes/edges. What is not clear is how these new nodes are initiated with respected to protein concentrations. This might have a strong influence on ligand diffusion dynamics and consequently the whole system dynamics. It would be good if this point was clarified and commented, for example in the "Materials and methods" section.

- The authors description of RA kinetics (Eqn 3) includes both a general degradation term (lambda) and a Cyp26b1 term (c1). It is unclear how the use of both terms is motivated. Do the authors have evidence that RA is degraded at a constant rate throughout the limb primordium in the absence of Cyp26b1? Is the uniform degradation rate necessary for the model to work?

- The 'cross-over' model the authors settle on (Eqns 11-12 etc) requires RA to act as a repressor of HoxA13. RA is normally considered to act as a transcriptional activator (via its receptors RAR etc). Moreover variants of the cross-over model (Model C, D, E) have RA activating Meis and repressing HoxA13. The authors need to comment on whether these observations are biologically motivated. Does it imply a repressor activated by RA that inhibits HoxA13 or do the authors think that RA is directly responsible for HoxA13 repression?

- The optimization technique the authors use does not provide any information on the parameter space of solutions. Sensitivity analysis or exploration of parameter space might provide more insight. It is notable that the authors do not discuss any of the parameter values. A superficial examination of the parameter tables in the supplementary data raises the possibility that there might be constraints on the relationships between some parameters. Analysis of the parameter values and the implications would strengthen the paper.

- It is unclear from the way the data are presented whether the final network the authors propose fits all the perturbation phenotypes. Model E performs well on wild-type and perturbation II, but it is not clear if it still performs well with perturbation I. Moreover, as a parameter had to be adapted to fit

perturbation III (a decay rate of one of the Hox genes is altered), it is unclear if this new network still fits the previous perturbations. The authors should clarify. The validation and refinement of the model using experimental perturbation data is a potential strength of this study but it is unclear whether the final model is the best fit for all conditions.

- The authors also conclude that their final model is "the most parcimonious" (Abstract) and it is "the only minimal GRN that could recapitulate" observed patterns (Discussion). However, this claim is not rigorously demonstrated as there is no systematic testing of all simpler topologies.

- The important conclusion from the authors analysis is that the gene regulatory network that they describe can act as both a levels-measuring and time-measuring circuit. This suggests an explanation to the controversy over previously proposed mechanisms - Uzkudun et al show that the same regulatory network can produce two different conceptual models. This idea has also become evident in other developing systems (for example Balaskas et al., 2012, Cell) and this emphasizes the central role of dynamics in gene regulatory networks. Extending the Discussion to make this point would strengthen the appeal of this paper.

Reviewer #2:

The manuscript focuses on mechanisms of pattern formation in the developing limb bud, in particular mechanisms by which different regions get specific as a function of exposure to patterning cues such as FGFs. It should be said, in addition, that the concepts explored in this manuscript - such as whether cell specification occurs as a result of exposure to different temporal durations of a given signal strength, different signal strengths for a fixed temporal duration, or differential exposure to 2 different signals - have implications beyond the limb bud system. The manuscript poses the problem very effectively, and the dialog between experiment and model is intriguing. In general, it is a potentially elegant approach to investigate different models that may drive developmental pattern formation. In addition, the experimental perturbation studies with the goal of model validation are laudable. That said, there are some questions about model formulation and the fitting approach that should be addressed.

Note that the timing mechanism proposed at the bottom of page 3 is distinct from the previous timing mechanism discussed. The former proposes that there are temporal windows during which a cell is competent to receive or act upon a signal (potentially due to epigenetic effects), whereas the previously discussed mechanism indicates that a cell competent to receive a signal makes a decision based upon the length of time it receives that signal. The two are conflated at the bottom of page 3.

In general, the downstream signaling pathways are highly abstracted, which involves a number of assumptions that should be explicitly stated. As just one example, different FGF ligands bind to different FGF receptors, which have different qualitative and quantitative extents of activation of downstream canonical pathways (which may or may not culminate in expression of their enzyme Cyp26b1). Lumping these together is reasonable for this high level model, but this assumption should be explicitly stated, preferably in the Results.

A rigorous, mostly like parameter fit to temporally evolving 2D data is interesting. That said, the investigators arbitrarily fix certain parameters and thus "set them aside" from the parameter fit, including production and decay constants and Hill parameters. Fixing these parameters must be better justified. Ideally, all parameters should be fit to the data, and sensitivity analysis should be conducted to provide statistical confidence intervals for each parameter (using many different parameter values in the parameter fitting search). This will help reveal whether parameter values are underdetermined by the fit. Also, if for example a Hill parameter is found to be equal to 2 plus or minus 5%, then their assumption of u=2 (or a higher value in the case of Hoxa11) would be justified. That is, parameter values shouldn't be arbitrarily chosen, especially ones that appear in highly nonlinear terms (such as Hill functions) that are likely to substantially impact model behavior.

On a related note, the Cross-Over model could better fit the data simply because it provides more adustable parameters. It is possible that other, arbitrary model formulations with a similar number of adjustable parameters could fit these data. Rigorous sensitivity analysis should be conducted, and

uniqueness of the Cross-Over model in quality of data fit should be examined. It is stated that only "minimal" gene regulatory networks were examined, for the goal of parsimony, but how comprehensive was the consideration of "minimal" and "more complex" model formulations, and how many and which models in each category were explored?

The inability of certain model formulations to account for experimental data is seemingly qualitative in the text (e.g. "both simulated Hoxa11 domains appeared as a curved stripe which is thicker in the middle than at the top of bottom" and "the real expression pattern is thinker and weaker in the middle.") This qualitative judgment call is unfortunately not substantiated by underlying quantitative results of model fit quality.

A superficial point perhaps, but it can be challenging to read a manuscript draft that is single spaced with small font.

In summary, this is an interesting blend of experiment and computation for an important problem in organismal development. The proposal that opposing gradients are necessary to generate this pattern is of course non-controversial in the developmental biology field, and an attempt to provide quantitative rigor to support this proposal is laudable. However, the rigor of model formulation and fitting should be improved.

Reviewer #3:

The manuscript by Uzkudun et al. proposes a modeling approach that tries to reconstruct the network topology underlying the patterning of the developing limb bud. They use an interesting and for this system novel reverse-engineering approach, where different proposed topologies are matched with 2D expression data following their previously published experimental and computational protocols (Marcon et al. 2011). In principle this is an interesting and potentially mechanistically insightful approach. However, there are a number of technical points the authors should address before an appropriate evaluation can take place.

Experimental:

- Why is an enzymatic labeling technique chosen over a fluorescence labeling technique? Provided images look saturated in the maximum expression region. Enzymatic labeling techniques are potentially highly non-linear. That is problematic even for relative measurements. No control is provided. What is the tolerance on saturation and non-linearities in labeling w.r.t. the model fits?
- The authors are correct in stating that absolute measurements are not needed. However, some sort of reference is needed in order to compare the expression levels in embryos of different developmental times. No control is provided. What is the tolerance on staging uncertainties and on systematic expression level fluctuations w.r.t. the model fits?

Modeling:

- No confidence in modeling approach is provided. These are large models, with over 20 parameters each, most of which are chosen ad hoc. The gradient descent method has to find the minimum in a 9-parameter space; that means the global minimum might never be found. Does that matter?
- How robust are the models w.r.t. to parameter perturbations? If for small parameter perturbations the model performs much worth an over-fitting situation might occur, which would hint at parameter fine-tuning--something rarely seen in biological systems at this level (four significant digits?).
- Are there qualitative differences in behaviors of different network topologies that could help gain confidence in the determined models?
- Can the authors provide out-of-sample predictions to validate their models? Can the correct models be found on a subset of data points (i.e. time points) and recapitulate the missing data?
- The final statement of the manuscript claims that a single network could be explained by two different models. Maybe. But another possibility is that this approach is unable to correctly discern between the two conceptual models. No measure or arguments are provided that would give the reader some confidence in the modeling approach allowing her/him to assess whether such statements are valid or not. Thus the utility of the approach is questionable.

A few minor points:

© European Molecular Biology Organization

- Crombach 2014 BioSystems should probably be cited somewhere
- In all figures make distinction between experiments and simulation clearer
- In text and figures decide between stage notation 10:22 or E10:22 (preferred); currently both are being used, which is confusing.
- On page 6, delete sentence starting: "As far as we know, ...for the first time..."

1st Revision - authors' response 25 March 2015

(see next page)

Point-by-point rebuttal for manuscript MSB-14-5882

New Title:
Data-driven modelling of a gene regulatory network for cell fate decisions in the growing limb bud.

Authors: Manu Uzkudun, Luciano Marcon, James Sharpe

24th March 2015

We would like to thank the reviewers for their very positive comments, and useful suggestions. We have been able to address all of their points, through a series of extra simulations, optimization and further analyses which are detailed in the Expanded Figures E1 to E6, and we believe this has indeed made the paper stronger.

Reviewer #1:

Uzkudun et al take a computational approach to test and refine a gene regulatory network that specifies proximodistal (PD) identity in the vertebrate limb. The authors use model optimization approaches, constrained by experimentally measured spatial patterns of gene expression, to identify models that interpret anti-parallel gradients of FGF and RA. The authors benchmark the model they derive against previous experimental perturbations of the tissue. A counter-intuitive observation from the model is that proximal morphogen (RA) appears to control the distal boundary of the target gene HoxA11 whereas the distal morphogen (FGF) controls its proximal boundary.

Overall this is a well performed study combining experimental and computational approaches. The conclusions are novel and will be of broad interest to developmental and systems biologists. The importance of the analysis is that the mechanism the authors propose reconciles two previous proposed models. These previous models were thought to be contradictory but Uzkudun et al show that an understanding of the dynamics of the gene regulatory network reveals that the two models can be implemented by the same dynamical system. The study nicely demonstrates the power of an interdisciplinary approach to provide new insight and describes the approach in a simple and accessible manner.

Nevertheless I think there are several issues that should be addressed to strengthen and clarify the study.

- The authors describe their approach as a "novel computation approach for reverse engineering gene regulatory networks". This might be a semantic issue and it doesn't detract from the value of the work, but I found this description misleading. First, it seems to imply that a network will be inferred from the data. Instead the authors are using model optimization techniques to parameterize differential equation models that describe the evolution of gene expression on a growing domain. There is no inference of topology and the 'reverse engineering' refers to the use of experimental gene expression patterns to develop target descriptions for the optimization. Second, I'm unclear what is 'novel' about the computational approach. The authors are using a gradient descent algorithm, which although not described in detail or referenced, appears to be a standard package. I would agree with the authors that bringing together the various aspects of the approach is novel, but I don't think this can really be labelled a 'novel computational approach'. I would suggest the authors use a different form of words to describe the approach in the Title

and Abstract, so that it does not lead readers to expect a different type of study. In addition the authors should provide more details of how the model optimization was performed.

We agree with the reviewer's point, and have changed the title and removed the first instance of the phrase "reverse engineering", replacing it with "parameter optimisation plus model selection". We keep the concept of reverse-engineering only later in the document, where we wish to emphasize that a collection of different topologies were tested, and the results actually determine which of these topologies gives the best fit to the data.
Addtionally, we have referenced correctly the gradient descent software used, in the materials and methods section.

- The authors place some emphasis on the idea that their work is the first "automated reverse-engineering... on a 2D growing/moving tissue". This may be the case. However the authors don't make clear why this is particularly challenging. Including growth does not appear to change the distance function(Eqn 6) used for scoring the optimization.

Indeed our phrasing was misleading. The distance function we used was not affected by the fact that the tissue is moving. We emphasize now that the main difference is the extra value of capturing real data in a growing/moving system, and therefore having more accurate and complete data to derive a model. The extra challenges came not from the distance function, nor from the optimization function, but from handling a dynamical model like this, which requires more computational power.

- Related to this the authors explain that the growth simulations involve refining a triangular mesh describing the tissue by adding new nodes/edges. What is not clear is how these new nodes are initiated with respected to protein concentrations. This might have a strong influence on ligand diffusion dynamics and consequently the whole system dynamics. It would be good if this point was clarified and commented, for example in the "Materials and methods" section.

Our method for solving PDEs on the dynamical triangular mesh was a rather involved work in its own right, which we previously published in Marcon et al. 2011. We now make clear that all details of the method should be found in that paper. The specific answer to the reviewer's question is that mass or amounts of proteins, are conserved – the contents of each triangle is split into the new re-meshed elements, and the new concentrations calculated (based on the areas of the triangles).

- The authors description of RA kinetics (Eqn 3) includes both a general degradation term (lambda) and a Cyp26b1 term (c1). It is unclear how the use of both terms is motivated. Do the authors have evidence that RA is degraded at a constant rate throughout the limb primordium in the absence of Cyp26b1? Is the uniform degradation rate necessary for the model to work?

A background decay rate for for RA, in addition to active degradation by Cyp26b1, has previously been suggested in the limb and in other systems (for example: "Sharp Developmental Thresholds Defined Through Bistability by Antagonistic Gradients of Retinoic Acid and FGF Signaling" (Goldbeter 2007), and "A quantitative analysis of the effect of all- trans-retinoic acid on the pattern of chick wing development". (Eichele 1985)

Taking these references into account we included in the model a background decay term

in addition to active Cyp26b1 degradation. Nevertheless, parameter sensitivity analysis (see Figure E6), reveals that this is not a relevant parameter.

- The 'cross-over' model the authors settle on (Eqns 11-12 etc) requires RA to act as a repressor of HoxA13. RA is normally considered to act as a transcriptional activator (via its receptors RAR etc). Moreover variants of the cross-over model (Model C, D, E) have RA activating Meis and repressing HoxA13. The authors need to comment on whether these observations are biologically motivated. Does it imply a repressor activated by RA that inhibits HoxA13 or do the authors think that RA is directly responsible for HoxA13 repression?

Although as the reviewer says, RA is known more as transcriptional activator, there is evidence in the literature that it can also act as a transcriptional repressor (for example: “Retinoic Acid Down-Regulates Aldehyde Dehydrogenase and Increases Cytotoxicity of 4-Hydroperoxycyclophosphamide and Acetaldehyde” (Moreb 2004), and “Retinoic acid down-regulates Tbx1 expression in vivo and in vitro.” (Roberts 2005). Regarding whether it is a direct or indirect repression, as our regulatory model is a “high level” description of the real interactions of the system, there is no explicit distinction between these options.

- The optimization technique the authors use does not provide any information on the parameter space of solutions. Sensitivity analysis or exploration of parameter space might provide more insight. It is notable that the authors do not discuss any of the parameter values. A superficial examination of the parameter tables in the supplementary data raises the possibility that there might be constraints on the relationships between some parameters. Analysis of the parameter values and the implications would strengthen the paper.

We agree that some serious extra analysis of parameter values was necessary, and have spent most of our effort over the last 3 months performing extensive extra analysis. In this regard, we have performed work in three main areas:

(1) Has our optimization found the global optimum in parameter space?
It is indeed important to determine how well the parameter values are constrained for a successful solution. Our numerical simulation is quite computationally heavy, and we therefore cannot perform large-scale parameter sweeps. However, we do have enough power to perform the parameter optimization process multiple times, each time starting from different initial values (ie. starting from different positions in parameter space). In this way we can determine whether the successful results always converge onto the same parameter values.
We have now performed this analysis for each of the initial models A, B, C and D. In each case we selected 27 different starting positions, chosen to be far from each other in parameter space. Using this approach we were able to confirm that model C is still the best model, and importantly, that there was a clear convergence of parameter values to the same position in parameter space for the successful runs. This is now highlighted in Figure 4j-l.

(2) Sensitivity analysis in the final model.
Related to the above, we also wish to know how sensitive the model is to changes in parameter values. For this we have performed a sensitivity analysis, in which each we measure the decrease in model fitness as each parameter is gradually increased or decreased. We then examined the differences in sensitivities to see if they make sense in the context of what we know about the model. The results and discussion are given in Figure E6.

(3) Meaningfulness of the parameter values.
Finally, we have also gone through all the parameters of the model to consider if the optimized values make sense in the context of this abstract model. Many of these parameters are relative with respect to each other, and some are more abstract than others (eg. the regulatory effect of one transcription factor on another is a high-level abstraction, while the diffusion constant of RA may be a direct biophysical measurement). But overall, within this context we see that the values used in the final model are all reasonable, and this is described in the legend for Figure E6.

- It is unclear from the way the data are presented whether the final network the authors propose fits all the perturbation phenotypes. Model E performs well on wild-type and perturbation II, but it is not clear if it still performs well with perturbation I. Moreover, as a parameter had to be adapted to fit perturbation III (a decay rate of one of the Hox genes is altered), it is unclear if this new network still fits the previous perturbations. The authors should clarify. The validation and refinement of the model using experimental perturbation data is a potential strength of this study but it is unclear whether the final model is the best fit for all conditions.

This is a good point, which we did not make clear enough. Indeed the final model (which is now called model F) has been tested on all the previous tests of the paper (against wildtype data, manipulations of RA and Meis levels). These "re-tests" have now been included explicitly in Figure E5.

- The authors also conclude that their final model is "the most parcimonious" (Abstract) and it is "the only minimal GRN that could recapitulate" observed patterns (Discussion). However, this claim is not rigorously demonstrated as there is no systematic testing of all simpler topologies.

Indeed we had not included every single possible simple topology. We have now done this (hence the reason for the re-naming/lettering of the models). We also now emphasize more clearly that the major question in the study is the regulation of the 2 Hox genes: Hoxa11 and Hoxa13. Regulation of Meis is already well-described in the literature cited in the introduction. This point is emphasized in Figure 1e, which shows which parts of the network we take as given (solid interactions). Consequently, there are 2 main inputs (RA and FGF), 2 main outputs (a11 and a13), and 4 possible regulatory links (dashed interactions in Figure 1e). Models A to D now cover all the 4 possible simplest networks (in which both output genes have at least one input).

We have also added another analysis in which we tested a new model (model X_0), which rather than being one of the simplest topologies, represents the "most complex" topology, ie. it contains all the possible regulatory links between the RA/FGF and the Hox genes. We optimized this "super-model" and then tested it by removing links (thus testing a series of intermediate models, which we have called X_1-X_4). In support of the previous approach, the links which are most important to maintaining a good score are indeed the 2 links of Model C. This new analysis is all in Figure E4. We have named this series of models as X_0 – X_4 so that they don't interfere with the flow of the story in the main text.

- The important conclusion from the authors analysis is that the gene regulatory network that they describe can act as both a levels-measuring and time-measuring circuit. This suggests an explanation to the controversy over previously proposed mechanisms - Uzkudun et al show that the same regulatory network can produce two different conceptual models. This idea has also

become evident in other developing systems (for example Balaskas et al., 2012, Cell) and this emphasizes the central role of dynamics in gene regulatory networks. Extending the Discussion to make this point would strengthen the appeal of this paper.

We were of course very happy to extend the discussion in the manner suggested.

Reviewer #2:

The manuscript focuses on mechanisms of pattern formation in the developing limb bud, in particular mechanisms by which different regions get specific as a function of exposure to patterning cues such as FGFs. It should be said, in addition, that the concepts explored in this manuscript - such as whether cell specification occurs as a result of exposure to different temporal durations of a given signal strength, different signal strengths for a fixed temporal duration, or differential exposure to 2 different signals - have implications beyond the limb bud system. The manuscript poses the problem very effectively, and the dialog between experiment and model is intriguing. In general, it is a potentially elegant approach to investigate different models that may drive developmental pattern formation. In addition, the experimental perturbation studies with the goal of model validation are laudable. That said, there are some questions about model formulation and the fitting approach
that should be addressed.

Note that the timing mechanism proposed at the bottom of page 3 is distinct from the previous timing mechanism discussed. The former proposes that there are temporal windows during which a cell is competent to receive or act upon a signal (potentially due to epigenetic effects), whereas the previously discussed mechanism indicates that a cell competent to receive a signal makes a decision based upon the length of time it receives that signal. The two are conflated at the bottom of page 3.

We did try to make the distinction (as the first idea referred to “duration of exposure to FGF”, while the second idea referred to “the age of the tissue”), however they may have seemed conflated because we also said “the idea of a timing mechanism has been revived”. We have now corrected this in the main text.

In general, the downstream signaling pathways are highly abstracted, which involves a number of assumptions that should be explicitly stated. As just one example, different FGF ligands bind to different FGF receptors, which have different qualitative and quantitative extents of activation of downstream canonical pathways (which may or may not culminate in expression of their enzyme Cyp26b1). Lumping these together is reasonable for this high level model, but this assumption should be explicitly stated, preferably in the Results.

This has now been made more explicit.

A rigorous, mostly like parameter fit to temporally evolving 2D data is interesting. That said, the investigators arbitrarily fix certain parameters and thus "set them aside" from the parameter fit, including production and decay constants and Hill parameters. Fixing these parameters must be better justified. Ideally, all parameters should be fit to the data, and sensitivity analysis should be conducted to provide statistical confidence intervals for each parameter (using many different parameter values in the parameter fitting search). This will help reveal whether parameter values are underdetermined by the fit. Also, if for example a Hill parameter is found to be equal to 2 plus or minus 5%, then their assumption of u=2 (or a higher value in the case of Hoxa11) would be

justified. That is, parameter values shouldn't be arbitrarily chosen, especially ones that appear in highly nonlinear terms (such as Hill functions) that are likely to substantially impact model behavior.

We agree with the reviewers' points here, and have made the following changes to address them:

(1) We now leave the Hill coefficients free for fitting (mu and mu') and the values we use now are the result of a full parameter optimization. Gratifyingly, they always converge to similar values for multiple different successful optimisations of Model C (1.25 and 3.77 respectively), which seem very reasonable.
(2) We give an explicit justification for the remaining fixed values (such as maximum production, decay and diffusion rates – apart from decay rates of RA and FGF which are still left free for fitting).
(3) We have also performed a parameter sensitivity analysis (as explained above in response to reviewer 1's comment). This indeed helps to show which parameter values are critical, and which are under-determined.
(4) We have also added a brief discussion of the results of the sensitivity analysis, and the justification for our choices (legend of Figure E6). This explains why one of our parameters is very insensitive (the background decay rate of RA is unimportant because its active regulation by Cyp26b1 has a dominant effect on the system). Indeed we have re-done the optimsation without this decay term, and see no change to the model.

On a related note, the Cross-Over model could better fit the data simply because it provides more adustable parameters. It is possible that other, arbitrary model formulations with a similar number of adjustable parameters could fit these data. Rigorous sensitivity analysis should be conducted, and uniqueness of the Cross-Over model in quality of data fit should be examined. It is stated that only "minimal" gene regulatory networks were examined, for the goal of parsimony, but how comprehensive was the consideration of "minimal" and "more complex" model formulations, and how many and which models in each category were explored?

To address this point we have added a new model (now called model D). We now emphasize more clearly that the major question in the study is the regulation of the 2 Hox genes: Hoxa11 and Hoxa13. Regulation of Meis is already well-described in the literature cited in the introduction. This point is emphasized in Figure 1e, which shows which parts of the network we take as given (solid interactions). Consequently, there are 2 main inputs (RA and FGF) and 2 main outputs (a11 and a13). Models A to D now cover all the 4 possible simplest networks (in which both output genes have at least one input). We now emphasize in the text that all 4 models (A to D) have the same number of adjustable parameters (because they all have 2 links from the upstream nodes of RA/FGF, to the downstream nodes a11/a13) – so model C cannot be the best simply though having more degrees of freedom.
Additionally, we have analysed a collection of more complex models (X_0 to X_5), which also addresses the question of which parameters (and which topological links) are the most important for the successful mechanism. All these extra results confirm our original conclusion, that the "crossed-over" links of Model C are the most important for a successful result.
Finally, and as discussed above, we have included a sensitivity analysis for the final Model F, and an explanation of why certain parameters are less sensitive than others.

The inability of certain model formulations to account for experimental data is seemingly qualitative in the text (e.g. "both simulated Hoxa11 domains appeared as a curved stripe which is thicker in the middle than at the top of bottom" and "the real expression pattern is thinker and weaker in the middle.") This qualitative judgment call is unfortunately not substantiated by underlying quantitative results of model fit quality.

> We thank the reviewer for highlighting a lack of clarity in our text, which we have corrected now. Our evidence for Model C being the best is indeed quantitative. Out of 27 otpimisations per model, Model C produces a quantitatively better fit, both in its difference score (2.41 compared to 2.53 for Model A) and also in the ease with which a successful model could be found (19/27 optimisations for Model C, versus 9/27 optimisations for Model A).
> The qualitative description to which the reviewer is referring is an intuitive explanation of why this is true. It is not the evidence, but rather the explanation – although it is presented as a hypothesis in the paper. We have tried to make this clear in the text now.

A superficial point perhaps, but it can be challenging to read a manuscript draft that is single spaced with small font.

In summary, this is an interesting blend of experiment and computation for an important problem in organismal development. The proposal that opposing gradients are necessary to generate this pattern is of course non-controversial in the developmental biology field, and an attempt to provide quantitative rigor to support this proposal is laudable. However, the rigor of model formulation and fitting should be improved.

> We thank the reviewer for these positive comments, and we believe we have indeed strongly improved the rigor of the analysis.

Reviewer #3:

The manuscript by Uzkudun et al. proposes a modeling approach that tries to reconstruct the network topology underlying the patterning of the developing limb bud. They use an interesting and for this system novel reverse-engineering approach, where different proposed topologies are matched with 2D expression data following their previously published experimental and computational protocols (Marcon et al. 2011). In principle this is an interesting and potentially mechanistically insightful approach. However, there are a number of technical points the authors should address before an appropriate evaluation can take place.

Experimental:

- Why is an enzymatic labeling technique chosen over a fluorescence labeling technique? Provided images look saturated in the maximum expression region. Enzymatic labeling techniques are potentially highly non-linear. That is problematic even for relative measurements. No control is provided. What is the tolerance on saturation and non-linearities in labeling w.r.t. the model fits?

> We thank the reviewer for pointing out this issue, which has allowed us to address it very strongly in the new version of the paper. Indeed, we agree that in-situ hybridization is a very non-linear method. The rationale for why our approach should work is that the information extracted about gene expression is primarily shape-information, rather than levels-information. Indeed questions about saturation and non-linearities are important.

To address this issue we have challenged our method by performing non-linear re-scaling of the expression data, to see if we still recover the same conclusion (that Model C is the best). We have filtered both the experimental data and the simulation results through a sigmoid Hill function. An extreme example would be a binarisation of the data, such that each pixel would be ON or OFF. Instead we chose a Hill coefficient of 4, such that the expression shapes become sharper, and the levels at any given pixel are explicitly altered in a non-proportional (non-linear) manner. When running the full optimsation of all 4 models (27 starting conditions for each) we still retain the original result, that Model C is the best.

To go one step further, we then altered the sigmoid filter (the effective threshold value) and re-ran all the optimisations a second time. Although the shapes are noticeably altered, and the levels are again altered in a non-linear manner, the overall conclusion remains unchanged: Model C is still the best.

All these extra runs of optimisations on re-scaled data are shown in Figure E2.

- The authors are correct in stating that absolute measurements are not needed. However, some sort of reference is needed in order to compare the expression levels in embryos of different developmental times. No control is provided. What is the tolerance on staging uncertainties and on systematic expression level fluctuations w.r.t. the model fits?

We believe that the extensive non-linear rescaling experiments described above address this issue as well.

Modeling:

- No confidence in modeling approach is provided. These are large models, with over 20 parameters each, most of which are chosen ad hoc. The gradient descent method has to find the minimum in a 9-parameter space; that means the global minimum might never be found. Does that matter?

We have now re-run the optimisations for each model 27 times, from different starting positions in parameter space. It is now clear that the parameters always converge for the successful runs (Figure 4j-l).

- How robust are the models w.r.t. to parameter perturbations? If for small parameter perturbations the model performs much worth an over-fitting situation might occur, which would hint at parameter fine-tuning--something rarely seen in biological systems at this level (four significant digits?).

This is a good point, which overlaps with the points of the other 2 reviewers. We have now performed a parameter sensitivity analysis, and explain why most parameters are very well-determined, and why one of them is not (lambda-R).

- Are there qualitative differences in behaviors of different network topologies that could help gain confidence in the determined models?

We believe that the most important qualitative differences in behavior are those which we tried to highlight in our discussion about the curvature of Hoxa11 boundaries in relation to the curvature of the RA and FGF gradients (now in Figure 4). This discussion (which reviewer 2 highlighted appeared as a “qualitative judgement call”) is very important, as it explains in qualitative terms why Model C is the best. We have tried to make this discussion clearer now. Briefly - of the 4 possible regulatory links, the 2 links of Model C are best because they allow the more curved shape of the FGF gradient to control the proximal a11 boundary, while the less curved shape of the RA gradient controls the distal a11 boundary (via a13).

- Can the authors provide out-of-sample predictions to validate their models? Can the correct models be found on a subset of data points (i.e. time points) and recapitulate the missing data?

We have followed precisely the reviewers suggestion here. We have removed almost half of the time points from the sequence of experimental data, and re-run the fitting procedure. The resulting model (shown in Figure E3) is indistinguishable from the previous results.

- The final statement of the manuscript claims that a single network could be explained by two different models. Maybe. But another possibility is that this approach is unable to correctly discern between the two conceptual models. No measure or arguments are provided that would give the reader some confidence in the modeling approach allowing her/him to assess whether such statements are valid or not. Thus the utility of the approach is questionable.

We see here from the reviewer’s comment that we have not been clear enough in this part of the text. The two conceptual models are distinguished by certain aspects of their behavior. These behaviours (measuring time or measuring levels) are not mutually-exclusive, and thus our goal here is not to determine which model “explains the network”. Our goal is to explore whether our network model exhibits one behavior, or the other, or both. The results show that our single model can indeed exhibit both behaviours, without changing any parameter values. In other words, the 2 ideas are only separable if you keep them very abstract and conceptual. As soon as you build a real/simple network, it cannot avoid exhibiting both behaviours.
We feel that this observation is important in the context of the long historical debate about proximo-distal patterning, as it suggests how both ideas could be true in the same system.

A few minor points:

- Crombach 2014 BioSystems should probably be cited somewhere

We think the reviewer may be thinking of Cromback 2012, in which reverse-engineering was performed from non-quantitative in-situ data, and we have indeed included this paper.

- In all figures make distinction between experiments and simulation clearer

We have tried to clarify this in the figures.

- In text and figures decide between stage notation 10:22 or E10:22 (preferred); currently both are being used, which is confusing.

This is a good point, which we have now corrected.

- On page 6, delete sentence starting: "As far as we know, ...for the first time…"

Our statement says: "As far as we know, this is the first case of automated reverse-engineering (parameter optimization plus model selection) on a 2D growing/moving tissue." We wish to keep this sentence, as we believe it is an important first in the field of developmental systems biology.

Overall we wish to thank all 3 reviewers for their comments. Addressing these issues has been a very positive experience, which has boosted the paper, and given us a stronger sense of the parameter space we're working in.

Pre-acceptance letter 21 April 2015

Thank you again for submitting your work to Molecular Systems Biology. We have now heard back from the three referees who were asked to evaluate your manuscript. As you will see below, the reviewers are satisfied with the modifications made.

Pending some minor editorial issues the study is now suitable for publication.

Thank you for submitting this paper to Molecular Systems Biology.

Reviewer #1:

I am satisfied that the authors have addressed the points I raised during the initial review.
I remain of the view that this is an interesting and important study. It suggests an elegant and convincing solution to a longstanding question in the field over whether temporal or gradient mechanisms pattern the proximodistal axis of the vertebrate limb. The approach combines experimental and computational work in a satisfying way and is clearly and accessibly described.

Reviewer #2:

The authors have addressed the concerns of this reviewer.

Reviewer #3:

While my previous concerns about the methods and approach have not been allayed, I do feel that the authors have provided enough additional evidence for their choice of the "right" model that should satisfy the community interested in this type of computation. This system under consideration in this case is known to be difficult to access experimentally and the authors should be applauded simply for trying to make quantitative progress, which is why it is fine to publish this study. In the end it is ultimately a matter of taste which kind of modeling approach to pursue, and only time will tell how far such an unconstrained approach, in which the number of fit parameters heavily outweighs the amount of available data, will lead us.
